# MicroRNA miR-20a-5p targets CYCS to inhibit apoptosis in hepatocellular carcinoma

**DOI:** 10.1038/s41419-024-06841-0

**Published:** 2024-06-27

**Authors:** Olaniyi Olarewaju, Yuhai Hu, Hsin-Chieh Tsay, Qinggong Yuan, Simon Eimterbäumer, Yu Xie, Renyi Qin, Michael Ott, Amar Deep Sharma, Asha Balakrishnan

**Affiliations:** 1 Department of Gastroenterology, Hepatology, Infectious Diseases, and Endocrinology, Hannover Medical School, Hannover, Germany; 2 AAV Gene Therapy Research Group, Research Beyond Borders (RBB), Boehringer Ingelheim Pharma GmbH & Co. KG, Biberach an der Riss, 88400, Germany; 3 Department of Hepatopancreatobiliary Surgery, the First Affiliated Hospital, Fujian Medical University, Fuzhou, 350005 China; 4 Department of Biliary-Pancreatic Surgery, Affiliated Tongji Hospital, Tongji Medical College, Huazhong University of Science and Technology, 1095 Jiefang Avenue, Wuhan, Hubei China; 5 Research Group RNA Therapeutics & Liver Regeneration, REBIRTH Center for Translational Regenerative Medicine, Hannover Medical School, Hannover, Germany

**Keywords:** Oncogenes, Cancer metabolism

## Abstract

Hepatocellular carcinoma is a primary liver cancer, characterised by diverse etiology, late diagnoses, and poor prognosis. Hepatocellular carcinoma is mostly resistant to current treatment options, therefore, identification of more effective druggable therapeutic targets is needed. We found microRNA miR-20a-5p is upregulated during mouse liver tumor progression and in human hepatocellular carcinoma patients. In this study, we elucidated the therapeutic potential of targeting oncogenic miR-20a-5p, in vivo, in a xenograft model and in two transgenic hepatocellular carcinoma mouse models via adeno-associated virus-mediated miR-20a-Tough-Decoy treatment. In vivo knockdown of miR-20a-5p attenuates tumor burden and prolongs survival in the two independent hepatocellular carcinoma mouse models. We identified and validated cytochrome c as a novel target of miR-20a-5p. Cytochrome c plays a key role in initiation of the apoptotic cascade and in the electron transport chain. We show for the first time, that miR-20a modulation affects both these key functions of cytochrome c during HCC development. Our study thus demonstrates the promising ‘two birds with one stone’ approach of therapeutic in vivo targeting of an oncogenic miRNA, whereby more than one key deregulated cellular process is affected, and unequivocally leads to more effective attenuation of HCC progression and significantly longer overall survival.

## Introduction

Hepatocellular carcinoma (HCC) is a leading cause of cancer-related deaths, worldwide [1]. HCC is a complex liver malignancy often accompanied by underlying liver disease. Poor in-depth understanding of complex molecular mechanisms of HCC, late diagnoses, coupled with its resistance to currently available first and second line treatment options, leave surgical resection and liver transplantation as the two most currently utilized avenues for prolonging survival [2]. Therefore, novel and more effective therapeutic targets for HCC are required.

Non-coding microRNAs (miRNAs), are small nucleic acids readily taken up by the liver, enabling their therapeutic application against HCC. MiRNAs affect diverse biological processes and pathways, and function post transcriptionally by binding to 3’UTRs of specific target mRNAs. MiRNAs may also function as oncogenes and tumor suppressors. To study the effect of potential oncogenic miRNAs in HCC development, we previously performed miRNA expression profiling and extensive in silico analyses [3] on liver tissues from different stages of liver tumor development and regression in a conditional transgenic c-Myc-driven mouse model of HCC [4, 5]. A master transcriptional regulator under normal physiological conditions, c-Myc is upregulated in several cancers, including in over 50% human HCCs. It is an important driver of malignant transformation [6]. We therefore used a conditional doxycycline(doxy)-regulated c-MYC-driven mouse model of liver cancer (LAP-tTA/TRE-MYC or LT2/MYC) in our study [3–5, 7]. Doxy removal from the diet activates c-MYC and initiates liver tumor development [4]. Doxy re-administration suppresses c-MYC, initiating tumor regression [5, 8]. These conditional mice enable controlled analyses of tumor progression and regression. Through our in silico analyses, we identified miR-20a-5p (miR-20a) as a significantly upregulated miRNA in developed liver tumors compared to normal livers and regressing tumors. MiR-20a belongs to the largely oncogenic miR-17-92 microRNA cluster [9, 10]. MiR-20a has been reported as both a potential oncogene and a tumor suppressor in various cancers, however, a clearer role in hepatocellular carcinoma (HCC) remains elusive.

Although miR-20a-5p has already been shown to variably play a role in HCC in previous studies, our work highlights the therapeutic relevance of targeting miR-20a-5p in vivo. In our proof-of-concept study, we investigated the effects of modulating miR-20a expression on HCC, using in-depth in vitro analyses in human hepatoma cells, with xenograft studies, and via in vivo analyses in two independent conditional transgenic mouse models of HCC - the LT2/MYC and LT2/RAS mice. We show for the first time, that miR-20a directly targets cytochrome c (*CYCS*), a key effector of ATP synthesis and apoptosis [11], and demonstrate that miR-20a affects both the metabolic and apoptotic functions of cytochrome c. Importantly, our study shows targeting oncogenic miR-20a-5p, in vivo, leads to significant reduction in tumor burden and increases survival, highlighting an unequivocal and promising therapeutic avenue for hepatocellular carcinoma.

## Results

### MiR-20a is upregulated in LT2/MYC liver tumors and in human HCCs

**Fig. 1:**
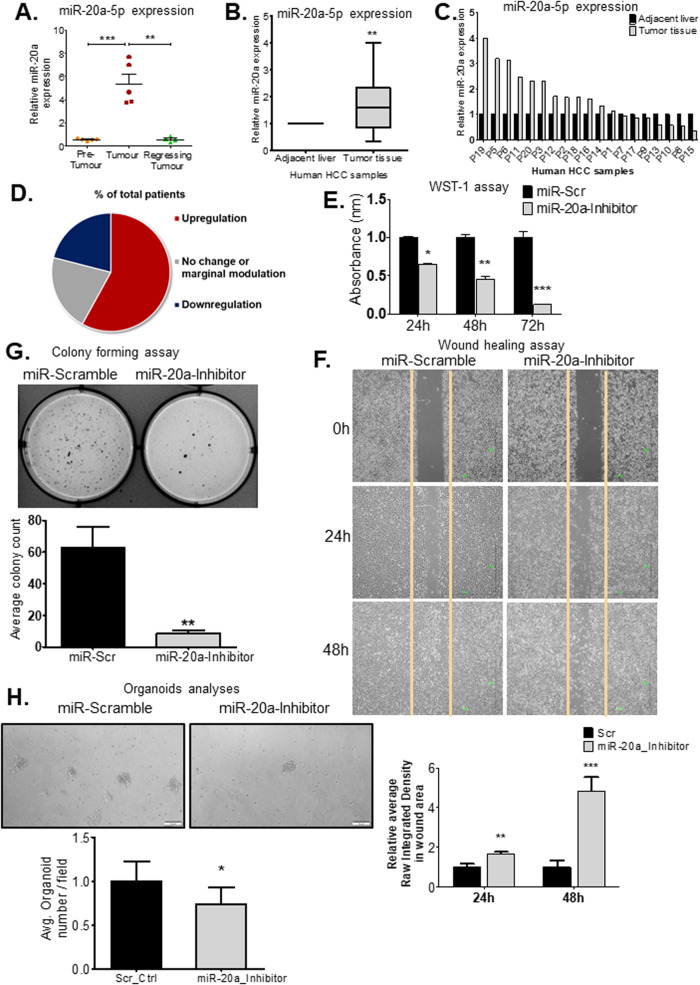
MicroRNA miR-20a-5p is a potential oncogenic miRNA, overexpressed during tumor progression. **A** RT-qPCR validation of miR-20a expression in liver tissue samples from stages of tumor development and regression. **, *P* < 0.001 and ***, *P* < 0.0001. **B–D** Comparison of miR-20a expression between tumor and adjacent liver tissue in a human HCC sample set (*n* = 19 patients). All 38 samples were run in triplicate (3 technical replicates) per miRNA/U6 internal control. **, *P* = 0.009. **E** WST-1 assay to determine differences in proliferation between miR-scramble and miR-20a-Inhibitor treated Huh7 cells (*n* = 6 independent biological replicates/group). *, *P* < 0.01; **, *P* < 0.001 and ***, *P* < 0.0001. **F** Wound-healing assay, shows a larger wound width correlating with impaired cell migration in the miR-20a-Inhibitor treated Huh7 cells compared to scramble treated cells at both 24 h and 48 h time points (*n* = 8 independent biological replicates per group and time point). **, *P* < 0.005 and ***, *P* < 0.0005. **G** Colony forming (Soft agar) assay to determine transforming ability of Huh7 cells treated with miR-Scramble and miR-20a-Inhibitor (data is from *n* = 3 independent biological replicates per treatment group from two independent experiments, i.e. *n* = 6 biological replicates/group).**, *P* = 0.002. **H** MiR-20a-inhibitor-treated LT2M cells form fewer liver tumor organoids than those treated with the scramble control. Graph shows average number of organoids/field/treatment group (Scramble = 21 fields, miR-20a-Inhibitor = 20 fields). (*n* = 3 biological replicates per treatment group). * *P* = 0.038. Unless otherwise specified, n= total number of independent biological replicates per group. Data in (**A**, **B**, **E-H**) are presented as mean ± SEM. *P* values were determined by two-tailed Student’s *t* test.

We had previously performed global miRNA expression profiling on livers at four different stages of tumor progression and regression—normal livers, early-stage pre-tumors, developed tumors, and early regressing tumors, from LT2/MYC mice [3]. Following extensive in silico analyses, we identified miR-20a as an oncogenic miRNA that was significantly and consistently upregulated in developed liver tumors and downregulated when tumors started to regress. Differential expression of miR-20a was confirmed in these mice livers via quantitative PCR (qPCR) (Fig. 1A). We also analysed miR-20a expression from 19 human HCC samples. Notably, miR-20a was upregulated in 58% (12/19 patients) of these samples compared to their adjacent non-tumor counterparts (Fig. 1B–D). Interestingly, human HCCs in the TCGA-LIHC dataset show higher miR-20a-5p expression in primary tumors compared to solid tissue normal samples (Supplementary Fig. S1A). We also observed that these HCC samples show a higher miR-20a-5p expression with increasing histological tumor grades (Supplementary Fig. S1B). This supports an oncogenic role for miR-20a in human HCCs.

### MiR-20a knockdown decreases proliferation, alters metabolism, and increases apoptosis in hepatocellular carcinoma cells

**Fig. 2:**
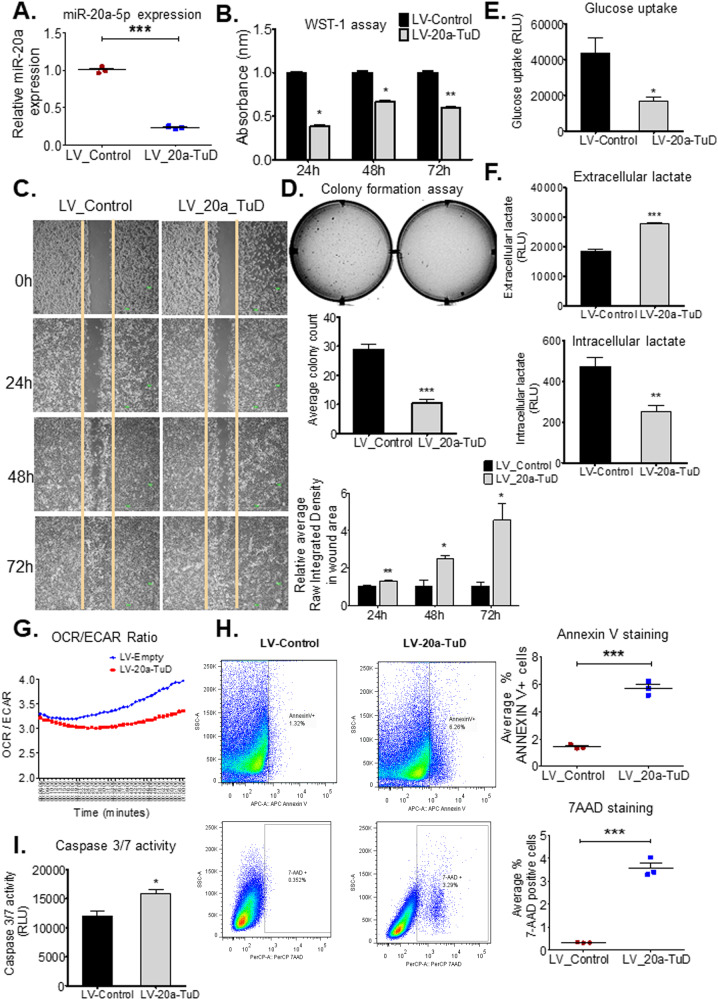
MiR-20a knockdown has anti-oncogenic effects on HCC cells. **A** QPCR quantification of miR-20a expression shows miR-20a downregulation following transduction of Huh7 with lentivirus LV-20a-TuD (*n* = 3 independent biological replicates/group). ***, *P* < 0.0001. **B** WST-1 assay shows decreased cell proliferation in LV-20a-TuD cells compared with control over 24 h, 48 h, and 72 h time points (*n* = 5 independent biological replicates/group). *, *P* = 0.05. **, *P* < 0.005. **C** Cell migration determined by wound healing assay shows a larger wound area, correlating with impaired cell migration in LV-20a-TuD cells at 24 h; 48 h and 72 h time points (*n* = 5 independent biological replicates per group and time point). *, *P* < 0.01 and **, *P* < 0.005. **D** Soft agar colony forming assay comparing the transforming ability of LV-20a-TuD cells with LV-Control Huh7 cells (*n* = 8). ***, *P* < 0.0001. **E** Glucose uptake was measured, and compared between LV-20a-TuD cells and control (*n* = 6). *, *P* = 0.01. **F** Lactate quantification shows increased extracellular accumulation (upper, ***, *P* < 0.0001), and decreased intracellular levels (lower, **, *P* = 0.0018) of lactate in LV-20a-TuD cells (*n* = 12). **G** OCR/ECAR ratio following OCR and ECAR assays shows a slightly lower ratio in the LV-20a-TuD cells compared with control cells plotted over time (in minutes) (*n* = 8). **H** Representative plots and graph from flow cytometry analyses showing ANNEXIN V positive cells (upper, ***, *P* < 0.0002) and 7-AAD positive cells (lower, ***, *P* < 0.0002) in LV-Control and LV-20a-TuD cells (*n* = 3). **I** Caspase 3/7 activity assay showing elevated activity in LV-20a-TuD cells (*n* = 5). *, *P* < 0.01. Unless otherwise specified, n= total number of independent biological replicates per group. Data in (**A–F**, **H**, **I**) are presented as mean ± SEM. *P* values were determined by two-tailed Student’s *t* test.

We used Huh7 human hepatoma cells to determine effects of miR-20a on hallmarks of cancer, in vitro. Huh7 was selected due to its intermediately high and significant miR-20a expression, making it amenable to miR-20a modulation studies (Supplementary Fig. S1C). Loss-of-function analyses were performed using 100 nM miR-20a inhibitor to achieve extensive, significant miR-20a knockdown (Supplementary Fig. S1D). A significant decrease in proliferation (Fig. 1E), migration over different time points (Fig. 1F), and colony forming ability (Fig. 1G) of Huh7 cells were observed following miR-20a inhibition, by WST-1 assays, wound healing assays, and soft agar colony forming assays, respectively. Inhibition of miR-20a-5p showed similar significant inhibitory effects when we performed these assays in an independent human hepatoma cell line, SNU-182 (Supplementary Fig. S2A–C). Additionally, we found that significantly fewer mouse HCC organoids developed from miR-20a-5p inhibitor-transfected LT2M cells compared to scramble control-transfected LT2M cells (Fig. 1H). LT2M cell line [12] was previously established from liver tumors from the LT2/MYC mouse model. We thus demonstrate miR-20a knockdown can also effectively decrease 3D tumor organoid development in addition to 2D in vitro culture. To ensure efficient, consistent, and long-term miR-20a downregulation, Huh7 cells were transduced with a lentivirus encoding miR-20a tough decoy (LV-20a-TuD). Tough decoy constructs are renowned for their efficacy in endogenous miRNA suppression [13]. We achieved over 75% miR-20a downregulation in LV-20a-TuD cells (Fig. 2A). Huh7-LV-Control cells, without the TuD [3] was used as a control. Significant decrease in LV-20a-TuD proliferation compared to LV-Control cells was observed with both WST-1 (Fig. 2B) and Click-iT EdU (Supplementary Fig. S3) assays. Additionally, wound-healing and colony forming assays, respectively, show impaired wound closure over different time points (Fig. 2C) and fewer number of colonies (Fig. 2D) in LV-20a-TuD cells. Our in vitro loss-of-function analyses using transient miR-20a-5p inhibitor, and stable Huh7-LV-20a-TuD cells, clearly show an oncogenic, tumor-promoting role for miR-20a.

We then investigated if miR-20a knockdown affects cellular energetics and metabolism. We quantified glucose uptake by determining accumulation of 2-Deoxy-d-glucose-6-phosphate (2DG6P), in vitro. Cells were given 2-Deoxy-d-glucose (2DG), a glucose analog, which is phosphorylated to yield 2DG6P and cannot be further metabolised by cells. Significant decrease in glucose uptake was seen in LV-20a-TuD cells (Fig. 2E). Lactate is utilised by cancer cells as a source for ATP production. Therefore, we analysed extracellular and intracellular lactate levels, to determine the effect of miR-20a knockdown on glycolysis and lactate utilisation. LV-20a-TuD cells showed higher extracellular lactate levels and lower intracellular lactate (Fig. 2F, upper and lower), indicating a decreased dependency on lactate as an alternative energy source. We also determined oxygen consumption rate (OCR) and extracellular acidification rate (ECAR). LV-20a-TuD cells showed a clear decrease in OCR and only a marginal decrease in ECAR, over time. LV-20a-TuD cells exhibited a lower OCR/ECAR ratio compared to LV-Control cells (Fig. 2G). These results indicate miR-20a knockdown shifts cellular bioenergetics by reducing metabolic fuel consumption and utilisation.

We then performed Annexin V and 7-AAD staining to evaluate the effect of miR-20a suppression on apoptosis. Both, Annexin V and 7-AAD (Fig. 2H, upper and lower) staining showed a significant increase in apoptotic cells following miR-20a knockdown. We also observed increased Caspase 3/7 activity in LV-20a-TuD cells by the Caspase 3/7 activity assay (Fig. 2I). Taken together, our in vitro data show knockdown of miR-20a leads to a decrease in cell proliferation and metabolic activity, and an increase in apoptosis.

### Cytochrome c is a novel direct target of miR-20a

We then analysed the molecular mechanism of the observed significant increase in apoptosis following miR-20a knockdown. We first determined expression of a subset of key apoptosis genes by qPCR, in LV-20a-TuD and LV-Control cells. No obvious significant difference was observed in BCL2 expression. Contrary to established models of apoptosis activation, BCL-xl was upregulated and anti-apoptotic MCL1 was significantly overexpressed in LV-20a-TuD cells (Supplementary Fig. S4A). MCL1 is a known target of miR-20a and is therefore expected to be upregulated following miR-20a knockdown [14]. Pro-apoptotic *BAD*, *BIM* and *BID* were significantly upregulated in LV-20a-TuD cells (Supplementary Fig. S4B). Expression of the main effectors of apoptosis, BAX and BAK were determined. BAX upregulation is required for mitochondria outer membrane permeabilization (MOMP), a critical event for apoptosis activation. While BAX was surprisingly downregulated, BAK was significantly upregulated by over two-fold in LV-20a-TuD cells (Supplementary Fig. S4C). This indicates BAK may play a compensatory role to promote cytochrome c release, enabling apoptosis [15].

**Fig. 3:**
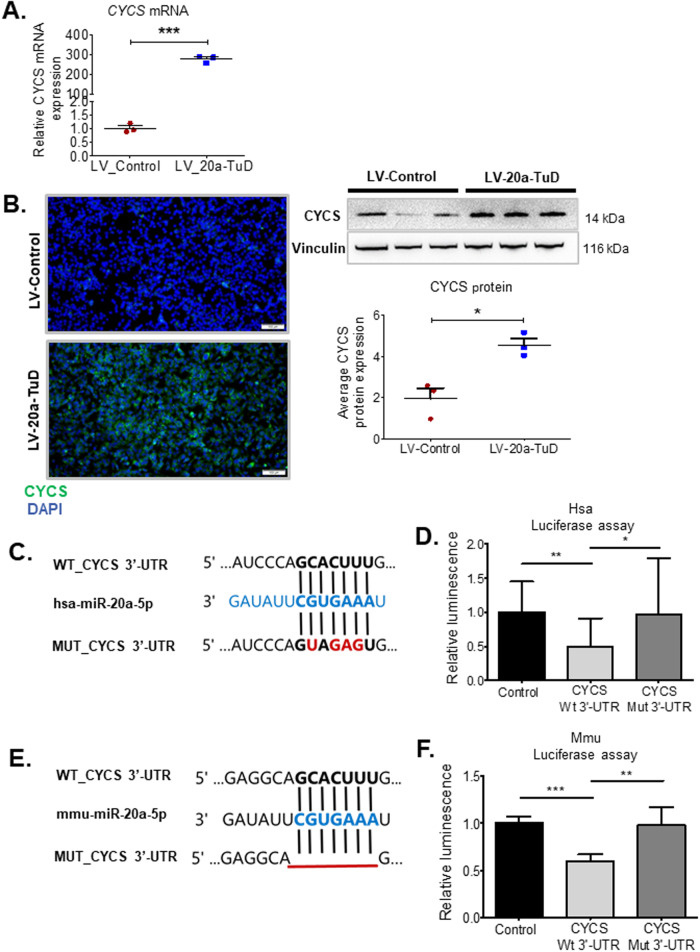
Identification of cytochrome c as a novel and direct target of miR-20a. **A** *CYCS* mRNA expression quantified by qPCR shows higher expression of *CYCS* in LV-20a-TuD cells (*n* = 3). ***, *P* < 0.0001. **B** Representative microscope images of cytochrome c immunofluorescence staining (left) and Western blot (right) show increased CYCS protein expression in LV-20a-TuD cells compared to LV-Control (*n* = 3). *, *P* < 0.02. **C** Schematic showing alignment of miR-20a with its wild-type binding site in the *Homo sapiens CYCS* 3’UTR sequence as well as the mutated binding site. **D** Validation of *CYCS* as a bona fide target of miR-20a by luciferase assay following cloning the human *CYCS* 3´UTR containing either the wild-type or mutated binding site, in the pmirglo vector (*n* = 5 per group). **, *P* < 0.005; *, *P* < 0.05. **E** Schematic showing alignment of miR-20a with its wild-type binding site in the mouse *Cycs* 3´UTR as well as the deleted binding site. **F** Validation of murine *Cycs* as a bona fide target of miR-20a with the luciferase assay following cloning of the mouse *Cycs* 3´UTR containing either the wild-type or deleted binding site (*n* = 5 per group). ***, *P* < 0.0001; **, *P* < 0.005. Unless otherwise specified, n= total number of independent biological replicates per group. Data are presented as mean ± SEM. *P* values were determined by two-tailed Student’s *t* test.

Next, we analysed genes further downstream in the apoptosis pathway and quantified mRNA expression of the cytochrome c gene (*CYCS*), which is released following MOMP to trigger activation of downstream effector caspases. Our qPCR showed a significant 273-fold increase in *CYCS* expression in LV-20a-TuD cells relative to LV-Control (Fig. 3A). This suggests the observed overexpression of CYCS is likely not due to upstream modulators, which normally initiate cytochrome c release. LV-20a-TuD cells also showed high CYCS expression by immunofluorescence staining (Fig. 3B, left) and a significant three-fold increase in CYCS protein expression (Fig. 3B, right) compared to LV-Control cells. *CYCS* has not been previously identified as a functional target of miR-20a with a biological significance.

To determine if *CYCS* is indeed a miR-20a target, we scanned the Homo *sapiens* 3’UTR sequence of *CYCS* for miR-20a binding sites, and found two almost identical binding sites. To determine whether *CYCS* is a functional bona fide target of miR-20a-5p, we performed luciferase assays. We cloned a fragment of *CYCS* 3’UTR harboring the binding sites into the pmirglo luciferase vector. Decrease in luciferase activity indicates binding of the miRNA to its target sequence. We found a significant decrease in luciferase activity in cells with CYCS 3’UTR compared to the Control 3’UTR vector. We then mutated the miR-20a-5p binding site on the *CYCS* 3’UTR. Disruption of the miR-20a-5p binding site on the CYCS 3’-UTR, inhibits binding of miR-20a-5p to CYCS, as indicated by no decrease in luciferase activity (Fig. 3C, D). Similarly, we also screened for the presence of miR-20a binding sites on the Mus *musculus* C*ycs* 3’UTR, and identified one miR-20a binding site. We then cloned a fragment of the mouse C*ycs* 3’UTR in the pmirglo vector and performed a luciferase activity assay to prove functionality of the binding site. Our result showed significant reduction in luciferase activity in cells with C*ycs* 3’UTR compared to Control 3’UTR vector, indicating miR-20a also directly targets *Mus musculus* C*ycs*. Further, deletion of the miR-20a binding site on the Mus *musculus* C*ycs* 3’UTR showed no significant change in luciferase activity compared to the control (Fig. 3E, F). Results from our luciferase assays confirm direct targeting of cytochrome c by miR-20a-5p.

To determine the effect of increased *MCL1* mRNA expression that we observed following miR-20a-5p knockdown, we inhibited endogenous *MCL1* in LV-20a-TuD cells with MCL1 siRNA. We then performed ANNEXIN V and 7-AAD to quantify apoptosis in these cells. MCL1 knockdown in LV-20a-TuD cells did not result in significant changes in number of either ANNEXIN V positive cells (Supplementary Fig. S5A) or in 7-AAD positive cells (Supplementary Fig. S5B). These results again indicate, the increased sensitivity to apoptosis we observed, likely results from an increase in *CYCS* expression following miR-20a inhibition. With these data, we show that cytochrome c is a bona fide target of miR-20a.

### Restoration of miR-20a results in downregulation of cytochrome c expression

**Fig. 4:**
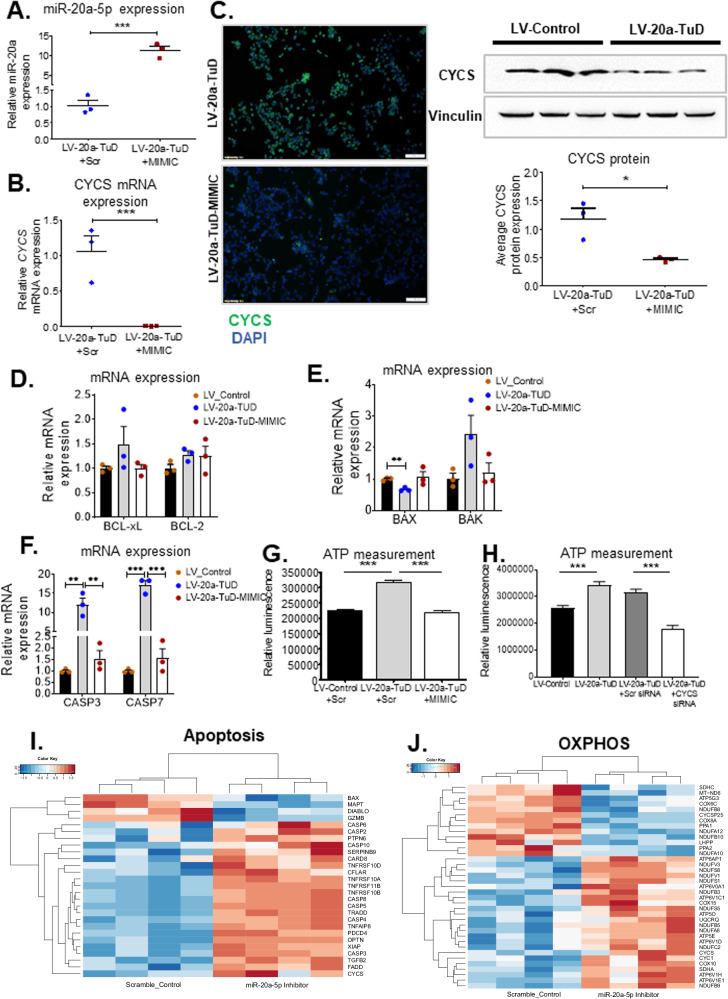
Restoration of miR-20a expression results in cytochrome c downregulation. QPCR data showing. **A** Overexpression of miR-20a in the LV-20a-TuD cells following the transfection of miR-20a mimic (*n* = 3). ***, *P* < 0.0002. **B** Complete downregulation of *CYCS* expression following miR-20a overexpression (*n* = 3). ***, *P* < 0.0002. **C** Representative microscope images of cytochrome c immunofluorescence staining (left) and Western blot (right) also show strong downregulation of CYCS protein when miR-20a is overexpressed (*n* = 3). *, *P* = 0.02. QPCR quantification of mRNA expression of (**D**) anti-apoptotic BCL-xL and BCL-2 (*n* = 3 independent biological replicates /group/gene) and (**E**) apoptosis effectors BAX and BAK (*n* = 3 independent biological replicates /group/gene). **, *P* = 0.002. **F** qPCR mRNA quantification of effector Caspases 3 and 7 shows deregulation of expression following miR-20a modulation (*n* = 3). **, *P* < 0.004; ***, *P* < 0.0004. **G**, **H** ATP production measured by luminescence assays in (**G**). Scramble-transfected LV-Control cells and LV-20a-TuD cells ± Scr or miR-20a mimic treatment (*n* = 10) ***, *P* < 0.0001, and in (**H**). LV-Control and LV-20a-TuD cells and LV-20a-TuD cells ±non-targeting (Scr) siRNA (*n* = 7) or Cyt c siRNA treatment (*n* = 9). ***, *P* < 0.0001. **I–J** Heat maps of (**I**) apoptosis and (**J**). OXPHOS genes following hierarchical clustering of gene expression data from Huh7 cells treated with either Scramble control or miR-20a-5p inhibitor. Clear differential regulation of apoptotic and OXPHOS related genes is seen between the two groups. n= number of independent biological samples/replicates per treatment group and number of separate samples sent for expression profiling. Data in (A, B, D–H) are presented as mean ± SEM. *P* values were determined by two-tailed Student’s *t* test.

To determine the effect of restoring miR-20a expression on *CYCS*, we performed rescue experiments by transiently transfecting miR-20a mimic in LV-20a-TuD cells. Following miR-20a mimic transfection, we found an 11-fold increase in miR-20a expression (Fig. 4A), a total suppression of *CYCS* mRNA expression (Fig. 4B), and downregulation of CYCS protein expression as seen by immunofluorescence (IF) staining (Fig. 4C, left) and western blot (Fig. 4C, right). These rescue experiments confirm miR-20a modulates CYCS expression.

We next determined if expression of BCL2 family members is altered following restoration of miR-20a in miR-20a mimic-treated LV-20a-TuD cells. Consistent with our initial observation, while there was no significant deregulation of BCL2, BCL-xl was slightly upregulated in scramble transfected LV-20a-TuD cells compared to scramble transfected LV-Control and mimic transfected LV-20a-TuD cells (Fig. 4D). Although the level of BCL-xl upregulation in scramble transfected LV-20a-TuD cells in Fig. 4D, was comparable to that observed in untreated LV-20a-TuD cells in Supplementary Fig. S4A, unlike the latter, it was not significant when compared to the corresponding LV-Control. Inspite of this difference, both sets showed a similar level of BCL-xl upregulation compared to their respective LV-Control groups. To investigate further, we also compared BCL-xl protein expression in LV-Control and LV-20a-TuD cells by western blot. BCL-xl protein levels did not significantly differ between the two groups (Supplementary Fig. S4D). This indicates that the marginal upregulation of Bcl-xl mRNA in LV-20a-TuD cells may not have a significant downstream biological effect in these cells. Pro-apoptotic BAX and BAK mRNA expression returned to levels comparable to LV-Control, following restoration of miR-20a expression (Fig. 4E). Taken together, our results indicate, direct regulation of *CYCS* by miR-20a is likely responsible for the significantly increased apoptosis observed in Huh7-LV-20a-TuD cells, following miR-20a knockdown.

Upon mitochondrial release, cytochrome c forms the apoptosome adapter complex with APAF1, resulting in caspase-9 activation. Decrease in *CYCS* expression following miR-20a-mimic treatment in LV-20a-TuD cells, led to a significant decrease in APAF1 and Caspase-9 protein expression, determined by western blot (Supplementary Fig. S6A, S6B). Thus, APAF1 and Caspase-9 expression were affected by cytochrome c availability. Caspase-3 and Caspase-7 expression were significantly upregulated following miR-20a knockdown in LV-20a-TuD cells and downregulated when miR-20a expression was restored (Fig. 4F). MiR-20a-mediated perturbation of CYCS therefore led to significant deregulation of key downstream apoptosis genes.

Apart from its central function in apoptosis, *CYCS* also plays a key role in the mitochondrial electron transport chain, in cellular respiration for ATP production. To understand whether miR-20a also affects the role of cytochrome c in bioenergetics in addition to its role in apoptosis, we determined if miR-20a-mediated downregulation of *CYCS* affected ATP production in cells. We measured and compared ATP levels in LV-Control, LV-20a-TuD and miR-20a-5p mimic-treated LV-20a-TuD cells. Our results show miR-20a knockdown increases ATP production, which decreases following miR-20a-5p mimic treatment (Fig. 4G). This was corroborated by decrease in ATP levels following siRNA-mediated *CYCS* knockdown in LV-20a-TuD cells (Fig. 4H). Further, global gene expression profiling on Huh7 cells treated with either scramble or with hsa-miR-20a-5p inhibitor showed upregulation of *CYCS* in miR-20a-inhibitor treated cells compared to scramble control. Additionally, apoptosis pathway genes and those involved in oxidative phosphorylation were differentially deregulated in miR-20a-inhibitor treated samples compared to scramble treated group, being largely upregulated in the former (Fig. 4I–J). Taken together, our extensive in vitro analyses reveal miR-20a is a potent modulator of *CYCS* and affects its’ roles in both apoptosis and oxidative phosphorylation.

### Inhibition of miR-20a attenuates tumor development in a xenograft model

**Fig. 5:**
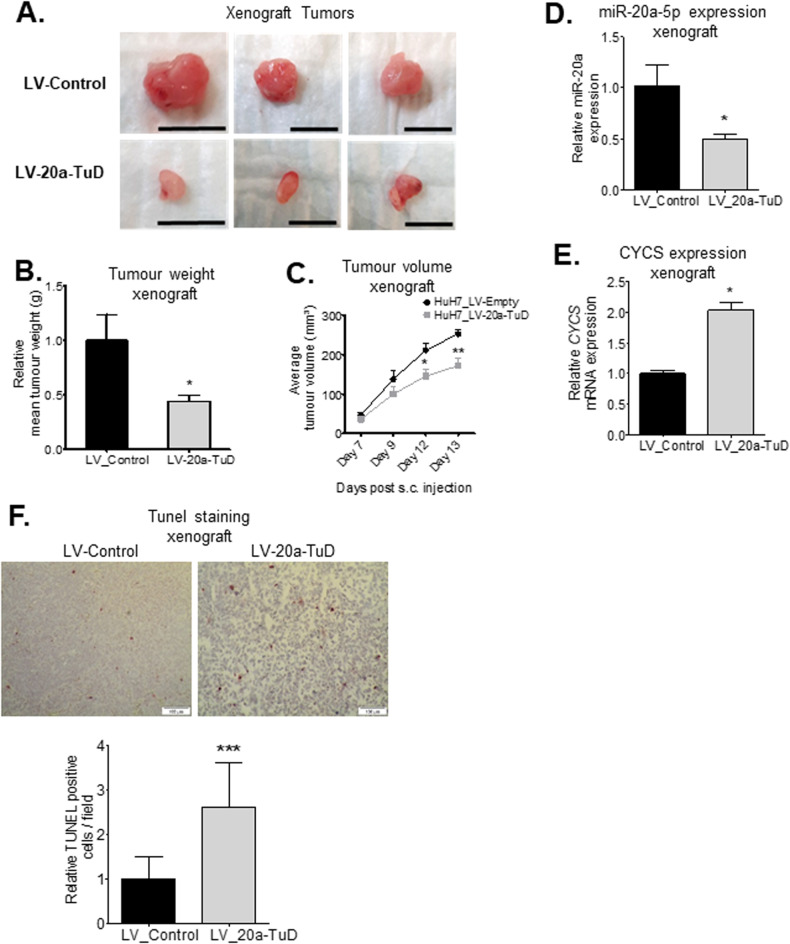
Knockdown of miR-20a attenuates tumor growth in a xenograft tumor model. **A** Representative images of xenograft tumors generated from LV-Control and LV-20a-TuD cells show a clear decrease in LV-20a-TuD tumor growth. **B** Graph shows significantly lower mean tumor weights of xenograft tumors from Huh7 LV-20a-TuD cells relative to those developed from Huh7 LV-Control and LV-20a-TuD cells in immunodeficient BALB/c nude mice (*n* = 5/group). *, *P* < 0.04. **C** Average tumor volume of all xenograft tumors per group are plotted at each time point of measurement, from the point of visible measurable tumor growth. *, *P* < 0.01 and **, *P* < 0.001. **D** qPCR quantification of miR-20a expression shows xenograft tumors developed from Huh7 LV-20a-TuD tumor tissues continue to show significantly lower miR-20a levels (*n* = 5/group). *, *P* < 0.002. **E** qPCR quantification shows significantly higher *CYCS* mRNA expression in LV-20a-TuD tumor tissues (*n* = 5/group). *, *P* < 0.001. **F** Representative images (20X magnification) of TUNEL staining on tissue sections from Huh7-LV-Control and Huh7-LV-20a-TuD xenograft tumors. Graph showing average TUNEL positive cells per field in Huh7-LV-20a-TuD xenograft tumors relative to the Huh7-LV-Control group. ***, *P* < 0.0002. n = number of mice per treatment group. Data are presented as mean ± SEM. *P* values were determined by two-tailed Student’s *t* test.

To determine if miR-20a knockdown affects xenograft tumor development, we injected 1.8 × 10^6^ LV-20a-TuD or LV-Control cells subcutaneously, into both flanks of adult female BALB/c Nu/Nu nude mice. MiR-20a knockdown attenuated xenograft tumor development, leading to smaller tumors (Fig. 5A), decreased tumor weight (Fig. 5B) and volume (Fig. 5C). We found miR-20a expression remained significantly lower in LV-20a-TuD tumors than in LV-Control tumors by over 50% (Fig. 5D), indicating miR-20a downregulation in these tumors was maintained over time. Conversely, LV-20a-TuD tumors showed a higher expression of *CYCS* (Fig. 5E) and significantly more TUNEL-positive apoptotic cells (Fig. 5F), compared to LV-Control tumors. Our data show miR-20a knockdown in a xenograft model attenuates tumor development, and increases apoptosis.

### In vivo knockdown of miR-20a attenuates tumor development in a mouse model of HCC

**Fig. 6:**
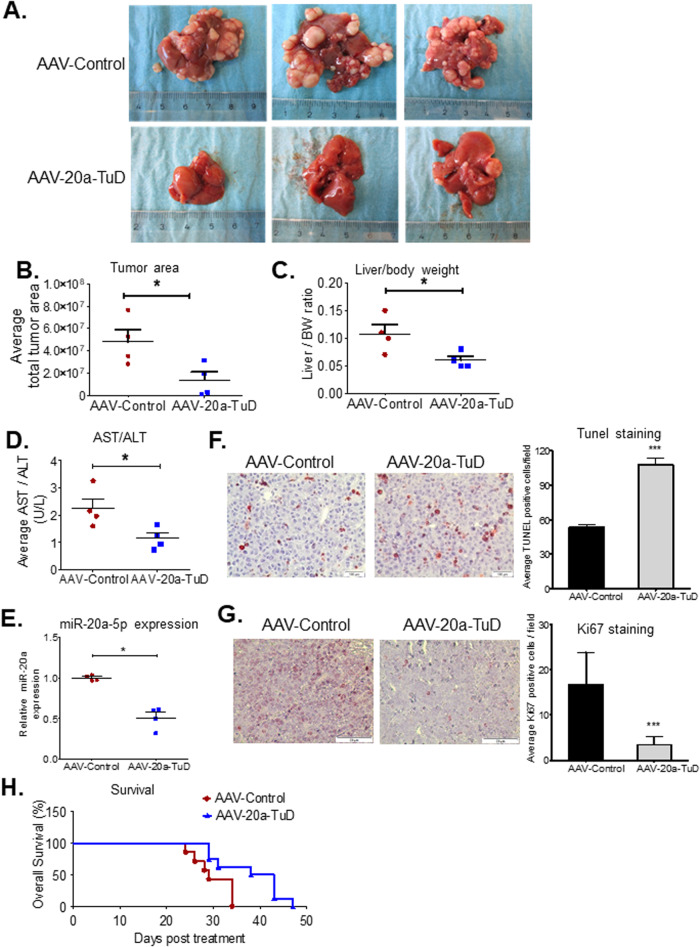
In vivo knockdown of miR-20a results in attenuation of tumor burden and prolongs survival in the LT2/MYC HCC mouse model. AAV-20a-TuD-treated MYC/LT2 mice show a clear attenuation of tumor development following AAV-20a-TuD treatment compared to AAV-Control mice as seen by (**A**). Different representative images of tumor-bearing livers from both groups. **B** Decreased relative total tumor area (*n* = 4), as calculated in Image J. *, *P* < 0.05. **C** Liver to body weight ratio (*n* = 4). *, *P* < 0.05, and (**D**). A decrease in AST/ALT ratio (*n* = 4). *, *P* < 0.05. **E** qPCR quantification of miR-20a expression in AAV-Control and AAV-20a-TuD treated MYC/LT2 mouse liver tumors (*n* = 4) confirms miR-20a knockdown in vivo. *, *P* < 0.05. **F**–**G** Representative images (×20 magnification) of liver tumor tissue sections from AAV-Control and AAV-20a-TuD treated MYC/LT2 mice following immunohistochemical staining for F. TUNEL (*n* = 30 fields/group) and (**G**) Ki67 (*n* = 40 fields/group), with corresponding graphs. Both, ***, *P* < 0.0001. **H** Kaplan-Meier survival analysis shows a significant increase in median survival of AAV-20a-TuD treated mice compared to AAV-Control mice (*n* = 6). Log-rank (Mantel-Cox) test was used to compare both treatment groups. *, *P* = 0.02. n= unless specified, number of mice per treatment group. Data in (**B**–**G**) are presented as mean ± SEM. *P* values were determined by two-tailed Student’s *t* test.

To determine the effects of in vivo knockdown of miR-20a on liver tumor development in a HCC mouse model, tumor-bearing LT2/MYC mice were intravenously administered 1.5*10^11^ AAV-Control and AAV-20a-TuD virus particles. Mice were sacrificed at 2-weeks post AAV injection. We observed a reduction in total tumor area (Fig. 6A, B) and a significant decrease in liver to body weight ratio (Fig. 6C) in AAV-20a-TuD-treated mice compared to the AAV-Control group. Mice receiving AAV-20a-TuD also had a lower AST/ALT ratio (Fig. 6D), consistent with reduction in overall tumor burden. Tumors from AAV-20a-TuD mice showed a 49% decrease in miR-20a expression compared to AAV-Control mice (Fig. 6E). To determine if increased susceptibility of tumor cells to apoptosis, contributed to the observed reduction in tumor burden, TUNEL staining was performed. AAV-20a-TuD injected mice showed significant increase in apoptotic cells (Fig. 6F). Additionally, Ki67 staining showed significant decrease in average number of Ki67 positive cells in AAV-20a-TuD injected mice (Fig. 6G). Both, the increase in apoptosis and decrease in cell proliferation seen in liver tumor tissues from AAV-miR-20a-TuD-treated MYC/LT2 mice were consistent with our in vitro analyses following miR-20a knockdown. To determine if AAV-20a-TuD treatment confers a survival advantage for tumor-bearing mice, we performed a survival study and compared survival curves using the Log-rank (Mantel-Cox) Test. AAV-Control treated mice showed a median survival of 28.5 days. AAV-20a-TuD administration led to a median survival of 40 days (Fig. 6H). Hence, a significant increase in overall survival of tumor-bearing MYC/LT2 mice was observed following AAV-20a-TuD treatment.

### HRAS-induced HCC model highlights the therapeutic effect of targeting miR-20a in vivo

**Fig. 7:**
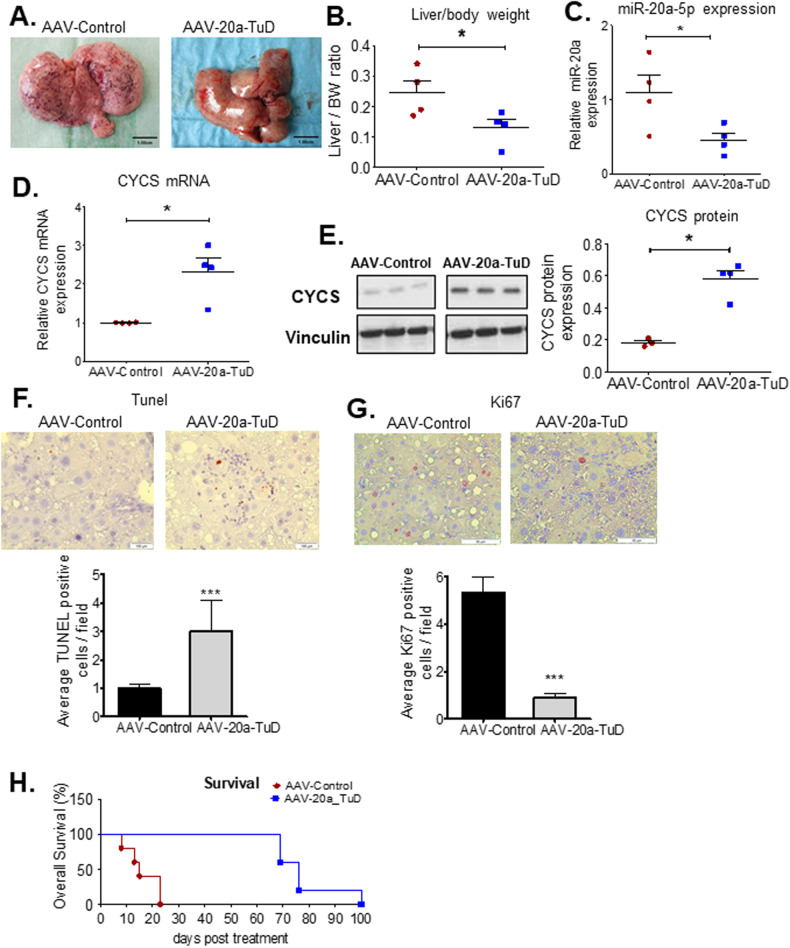
Depletion of miR-20a significantly attenuates tumor burden and prolongs survival also in the LT2/RAS HCC mouse model. **A** Representative gross images of LT2/RAS mice liver tumors from AAV-Control and AAV-20a-TuD treated mice showing an attenuation of tumor development following AAV-20a-TuD treatment. This is also reflected in (**B**) decreased liver/body weight ratio (*n* = 4). *, *P* < 0.05. qPCR analyses of liver tumor tissues from AAV-Control and AAV-20a-TuD treated LT2/RAS mice (*n* = 4) to determine expression of (**C**). miR-20a *, *P* < 0.05 and (**D**). *Cycs* levels (*n* = 4). *, *P* < 0.05. **E** Western blot shows significant increase in CYCS protein expression in AAV-20a-TuD mice (*n* = 4) compared to AAV-Control mice (*n* = 3). *, *P* < 0.01. **F** Representative images (20X magnification) of TUNEL stained liver tumor tissue sections and the graph showing average TUNEL positive cells per field in AAV-20a-TuD mice relative to the AAV-Control group (*n* = 4 mice/group, 12 fields counted per mouse per group). ***, *P* = 0.0002. **G** Representative images (×20 magnification) of Ki67 stained liver tumor tissue sections from the AAV-Control and AAV-20a-TuD treated mice with the graph showing average Ki67 positive cells per field (*n* = 4 mice/group, 10 fields counted per mouse / group). ***, *P* < 0.0001. n= number of mice per treatment group. **H** AAV-20a-TuD treated LT2/RAS mice show a significant increase in median survival compared to AAV-Control mice, as determined by Kaplan-Meier survival analysis (*n* = 5 mice per group) using the Log-rank (Mantel-Cox) test to compare both treatment groups. **, *P* = 0.0027. Data in (**B**–**G**) are presented as mean ± SEM, two-tailed Student’s *t* test.

To further evaluate the effect of in vivo targeting of miR-20a on HCC, we investigated this in a second independent mouse model of HCC - LT2/RAS mice. Our results show AAV-mediated miR-20a knockdown in LT2/RAS mice also leads to significant attenuation of tumor development (Fig. 7A, B). AAV-20a-TuD treated LT2/RAS mice show significantly lower miR-20a expression compared to the AAV-Control group (Fig. 7C). Concurrently, we observed a significant increase in cytochrome c mRNA (Fig. 7D) and protein (Fig. 7E) expression following miR-20a knockdown, in AAV-20a-TuD treated mice. A significant increase in apoptotic cells (Fig. 7F) and significant decrease in cell proliferation (Fig. 7G) were also observed in AAV-20a-TuD treated RAS/LT2 mice, by TUNEL and Ki67 staining, respectively. To determine whether in vivo miR-20a inhibition affects survival in RAS/LT2 mice, we performed a survival study. Our results show AAV-20a-TuD treated RAS/LT2 mice have a significant survival advantage with a median survival of 76 days, compared to AAV-Control treated mice with a median survival of 15 days (Fig. 7H). (Log-rank (Mantel-Cox) Test *p* < 0.0027).

**Fig. 8:**
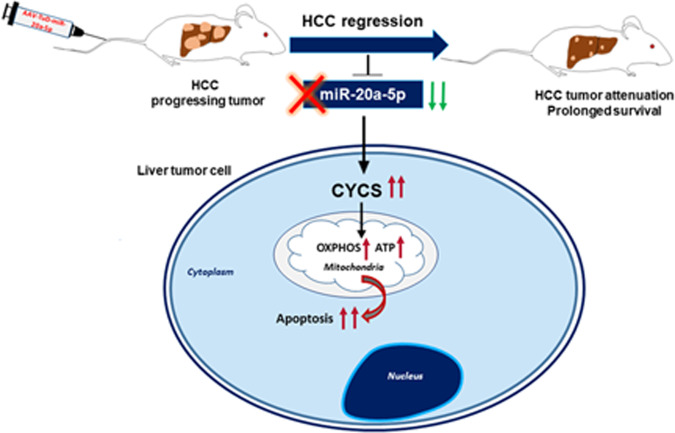
Schematic summarizing the primary findings of our study, shows in vivo inhibition of miR-20a-5p has a promising therapeutic inhibitory effect on HCC development. MiR-20a-5p inhibition increased expression of its direct target, CYCS, thereby leading to an increase in apoptosis and initiating a metabolic shift within tumor cells.

Taken together, our extensive in vitro studies, xenograft, and in vivo analyses using two independent mouse models of HCC, highlight the potent therapeutic effect of targeting oncogenic miR-20a-5p. Our study shows for the first time, that miR-20a targets cytochrome c, and affects both its function as a key regulator of apoptosis and in oxidative phosphorylation for ATP production. Importantly, through this study, we have demonstrated the efficacy of miR-20a inhibition in suppressing tumor development by targeting the multifunctional gene, CYCS (Fig. 8). This offers a highly effective approach for HCC treatment.

## Discussion

In this study, we investigated therapeutic targeting of oncogenic miR-20a in HCC, in-depth. MiR-20a is involved in progression and metastasis in many cancers [16–19]. We found that knocking down oncogenic miR-20a-5p in vitro significantly affects classical cancer hallmarks, including cell proliferation, invasion, colony formation and apoptosis. We used various experimental approaches to study the effect of miR-20a-5p modulation on these classical cancer hallmarks. For example, using the EDU assay, we found a small but significant difference in cell proliferation following miR-20a-5p knockdown. Additionally, the WST1 assay showed a clear decrease in cell proliferation over different time points in the miR-20a-5p knocked down cells, thereby supporting the EDU results and indicating a biologically relevant effect.

We show oncogenic miR-20a significantly contributes to apoptosis evasion in HCC. It is thus possible that the significant decrease in cell migration we observe following miR-20a-5p knockdown could be in part attributed to increased cell death. However, potential target genes of miR-20a-5p that are more directly involved in preventing invasion or cell proliferation may also be affected by miR-20a-5p knockdown. For example, miR-20a-5p promotes invasion and metastasis in colorectal cancer by inhibiting Smad4 [17] and promotes proliferation of non-small cell lung cancer by targeting the potent tumor suppressor, PTEN [20].

Due to our observed significant effect of miR-20a-5p on apoptosis evasion, our study focused on analysing this interesting observation further. We identified for the first time, cytochrome c, as a novel direct target of miR-20a, when elucidating the molecular mechanism of miR-20a-mediated apoptosis inhibition in HCC. Encoded by *CYCS*, cytochrome c is a multifunctional protein [21] with key roles in both apoptosis and as an electron transporter in the electron transport chain, for ATP synthesis via oxidative phosphorylation. We found hepatoma cells reduced their sensitivity to apoptosis by miR-20a-mediated direct targeting of *CYCS*.

Dynamic interactions and regulation of the balance between pro- and anti-apoptotic genes occur to initiate the apoptotic cascade [22, 23]. Surprisingly, we found anti-apoptotic BCL2, BCL-xl, and MCL1 upregulated in LV-20a-TuD cells. MCL-1, a known direct target of miR-20a, inhibits MOMP and mitochondrial cytochrome c release. However, despite an increase in anti-apoptotic Mcl1 in LV-20a-TuD cells, we still saw significantly higher apoptosis. This clearly indicates the increased sensitivity to apoptosis we observe following miR-20a knockdown, resulted downstream, via de-repression of CYCS, its direct target. Although Bax expression decreased in LV-20a-TuD cells, apoptosis effector BAK was upregulated. Bak translocation is required to trigger MOMP and cytochrome c release [24]. Overlapping roles of Bax and Bak in apoptosis regulation [25] could explain a compensatory significant increase in Bak. In contrast to variable expression of the BCL-2 family upstream of CYCS, we found significant upregulation of proteins downstream of CYCS in the apoptotic pathway. Our results indicate apoptosis can also be triggered via targeted inhibition of miR-20a, causing CYCS upregulation.

Cytochrome c also plays a very essential role in the electron transport chain for ATP production via oxidative phosphorylation. We found miR-20a-mediated downregulation of *CYCS*, decreased ATP production in cells and increased glucose uptake. In contrast, tough decoy-mediated miR-20a knockdown increased CYCS expression, thereby increasing ATP levels. However, when these cells, with low miR-20a, were treated with *CYCS* siRNA, ATP consequently decreased. Importantly, apoptosis is an energy demanding biological process, which requires ATP for apoptotic progression, including Apaf-1 oligomerization and subsequent caspase activation [26, 27]. Elevation of ATP is a necessary part of apoptotic cell death [28] and enables apoptosis preventing a switch to necrosis [29, 30]. It is therefore likely that both ATP levels and apoptosis increase following CYCS upregulation.

Under physiological conditions, glycolysis and OXPHOS, are tightly coupled processes and an example of a molecular interconversion system [31]. Increased cytochrome c and the consequently higher ATP production we observe following miR-20a knockdown, therefore, also likely leads to a shift away from glycolysis, towards OXPHOS. This is further supported by our observation of decreased glucose uptake and lower intracellular and higher extracellular lactate levels in 20a-TuD cells. Oncogenic miR-20a-mediated *CYCS* knockdown could be a mechanism adopted by cancer cells in shifting from OXPHOS to a more glycolytic phenotype. Thus, miR-20a regulates mitochondrial respiration and ATP production by targeting the cytochrome c gene.

We determined whether in vivo targeting of miR-20a has a therapeutic effect on HCC, by AAV-mediated delivery of miR-20a tough decoy in two different transgenic mouse models of HCC. In these models, liver-specific activation of either c-Myc or HRASV12 oncogenes lead to different subtypes of HCC. Nodular c-Myc-driven liver tumors consist of poorly differentiated cells and resemble a less differentiated, highly proliferative variant of human HCC and hepatoblastoma. In contrast, HRASV12-driven liver tumors are not nodular, have large cells, and resemble human HCC [4]. Our in vivo studies using mouse models with very different types of liver tumors, clearly showed miR-20a knockdown attenuated tumor development and significantly improved overall survival in both models. MiR-20a depletion in vivo results in loss of its inhibitory effect on *CYCS*, and increases CYCS expression, indicating miR-20a also targets *CYCS*, in vivo.

Cancer cells can manipulate the cellular apoptotic machinery and acquire resistance to cell death signals [32–34]. Therefore, modulation of the apoptotic pathway has been explored for cancer therapy. These include targeting anti-apoptotic proteins such as BCL-2 [35] to predispose cancer cells to apoptosis, or modulating pro-apoptotic BH-3 domain only proteins [22, 36, 37], and apoptosis effector BAX, to increase sensitivity of cancer cells to apoptosis [38].

Direct delivery of CYCS protein into tumor cells, aimed at activating caspases and inducing apoptosis, has been attempted with variable effectiveness [39–42]. Hence, it is relevant to explore more effective approaches, via endogenous induction and release of CYCS. Our results indicate in vivo knockdown of miR-20a is an effective therapeutic strategy, leading to increased endogenous CYCS. Therapeutic modulation of miRNAs for liver cancer treatment takes advantage of the liver’s ability to readily take up small nucleic acids. This approach has shown promise in ours and others’ previous studies [3, 4, 43].

In conclusion, we elucidated the oncogenic role of miR-20a in promoting HCC progression. We identified CYCS as a novel target of miR-20a. Targeting miR-20a, upregulates CYCS, and therefore induces apoptosis and reprograms cellular bioenergetics in cancer cells. This has therapeutic implications. MiR-20a inhibition significantly attenuates tumor development, and prolongs survival of tumor-bearing mice, thus showing promising therapeutic potential in HCC intervention.

## Materials and methods

### Animal experiments

Animal experiments were approved by the local state authority (Niedersächsisches Landesamt für Verbraucherschutz und Lebensmittelsicherheit; Acceptance number: 19-3282). Legal and ethical requirements and the 3Rs (Replacement, Refinement and Reduction of Animals in Research), were followed when planning and performing experiments. Animals were housed in MHH-Twincore animal facility under daily care of animal caretakers and close monitoring by experimenters. Animals were fed *ad libitum* on either standard feed or doxycycline-containing feed with unrestricted water access.

The minimum number of mice for each group in each experiment, was four. Based on our previous experience with all in vivo models in this study, and keeping with the 3Rs, we selected the most suitable minimal sample size. Animals were selected randomly in our animal experiments. However, all groups had animals of comparable ages and the same gender or equal representation of both genders, where applicable. All in vivo experiments were analysed in a blinded manner, especially during sample collection and downstream analyses.

#### Xenograft experiments

10-12 weeks old female immunodeficient BALB/c nude mice (CAnN.Cg-Foxn1nu/Crl, Charles River) were subcutaneously injected on both flanks with either 1.8 ×10^6^ Huh7-LV-Control or HuhLV-20a-TuD cells. Once visible subcutaneous tumors developed, they were measured until a maximum tumor volume of 250mm^3^ was reached for at least two mice in the experiment, as per the end-point stated in our animal protocol recommendations. Mice were sacrificed and tumor tissues were collected for downstream analyses.

#### In vivo HCC experiments

To determine effects of oncogenic miR-20a knockdown on HCC development, in vivo, two independent transgenic conditional doxycycline-regulatable mouse models of HCC were used in both short-term and survival analyses. Adult, male LT2/MYC mice [5] taken off doxy-containing diet for 6-7 weeks were injected intravenously with 1.5*10^11^ AAV8-miR-20a-TuD or AAV8-Control virus particles, for short-term and survival studies. As a second in vivo model, female and male LT2/RAS mice [3] were used. AAV8-miR-20a-TuD and AAV8-Control viruses were injected in LT2/RAS mice 4 weeks after doxy withdrawal. Animals in short-term experiments from both HCC models were sacrificed two weeks following AAV injections. Mice in survival studies were monitored daily and sacrificed at the health status endpoint recommended in our animal protocol.

### MicroRNA expression profiling

Our in silico analyses to identify oncogenic miRNAs are based on previously performed and described global microRNA expression profiling [3]. Gene Expression Omnibus accession numbers for miRNA expression array data (Exiqon) is GSE152920.

### Gene expression profiling

Global mRNA microarray profiling was performed on DNase treated total RNA from Huh7 cells treated with miR-20a-5p inhibitor or Scramble control. Microarray data generated for the scramble control treated Huh7 cells has already been submitted to GEO (GSE152950) and published with another miRNA also analyzed in this array [4]. The data from the miR-20a inhibitor treated cells was also generated in the same experiment, however, this was not published in the previous study, and will now be appended to the same GEO dataset with a new accession number (GSE270068). Total RNA was extracted with the mirVana™ miRNA Isolation Kit (ThermoFisher Scientific) and DNase treated with the Ambion DNase I (RNase-free) kit (ThermoFisher Scientific). The Genome Analytics core facility at the Helmholtz Center for Infection Research (HZI), Braunschweig, Germany performed quality control with an Agilent 2100 Bioanalyzer (Agilent Technologies, Santa Clara, CA) and microarray profiling using the Agilent Whole Human Genome Oligo Microarrays (G4112A) platform. Background subtracted Processed Signal intensities were obtained using the Feature Extraction Software 10.5 (Agilent). Raw data were further analyzed using R package “Limma”. Raw data were log2 transformed and quantile normalized.

### In silico analyses

Cluster and TreeView programs were used to perform hierarchical clustering and to generate heat maps for the apoptosis and oxidative phosphorylation gene lists from the mRNA expression profiling array data from miR-20a inhibitor and scramble treated Huh7 cells. Graphpad, Prism was used to generate the graphs and perform the Kaplan-Meier analyses. Image J was used to calculate tumor area in the MYC/LT2 mice, and to quantify Ki67 and TUNEL positive cells following the respective staining on cells and from tissues from all experimental models.

### MicroRNA and gene expression analyses

Total RNA was extracted using the mirVANA miRNA isolation kit (ThermoFisher), and DNase treatment (Ambion DNase I) (RNase-free) was performed as per the enclosed kit protocols. The microRNA reverse transcription kit (Applied Biosystems, ThermoFisher Scientific) along with the specific TaqMan miR-RT probes for hsa-miR-20a-5p and the internal control U6 snRNA were used for miR-RT reactions. Their respective TaqMan (TM) probes were used with the Universal TaqMan PCR Mastermix (Applied Biosystem, ThermoFisher Scientific) for the qPCRs. To determine gene expression levels, total cDNA was synthesized with the iScript cDNA synthesis kit (BioRad). Specific SYBR Green qPCR compatible primer pairs for target genes of interest were designed (Supplementary data Table. S1) and used with the SYBR Green PCR Mastermix (Applied Biosystems) and cDNAs to set up the SYBR Green qPCRs. Both TaqMan and SYBR Green qPCRs were performed on a Roche LightCycler II machine, and relative gene expression was calculated using the 2^-DDCT method.

### Cell Culture and transfections

Huh7 (CLS, Cell Lines Service GmbH) is a human hepatoma cell line, which we extensively used for our in vitro and xenograft studies. To confirm our data from our initial in vitro studies in Huh7 cells, we also performed these experiments in SNU-182, an independent human hepatoma cell line. Both cell lines were cultured in Dulbecco’s modified Eagle’s medium (DMEM, 4.5 g/ml glucose), supplemented with heat-inactivated 10% fetal bovine serum (FBS) and 1% Penicillin/Streptomycin antibiotics. Cells in culture were maintained in an incubator at 37 °C with 5% CO_2_. For in vitro assays, we seeded 1 × 10^4^ to 1.5 × 10^4^ cells in 96-well plates, 5 × 10^4^ cells in 12-well plates, 1.5 × 10^5^ cells in 6-well plates, and 1 × 10^6^, 2 × 10^6^ and 3 × 10^6^ cells were seeded in T75 cm^3^, T175cm^3^ and T225cm^3^ flasks, respectively.

#### Transient transfections

For in vitro miR-20a modulation experiments in Huh7 cells, 50 nM or 100 nM of either miR-20a mimic, inhibitor, and miR-negative controls (Scramble) were transiently transfected using Dharmacon 4 as the transfection reagent. All were purchased from Dharmacon, Horizon Discovery. For CYCS and MCL1 modulation, the respective On-Target Plus SMARTpool siRNAs at 50 nM and 100 nM, respectively, and negative ON-TARGETplus Non-targeting Pool control were used (Dharmacon, Horizon Discovery). Dharmacon 4 was used as the transfection reagent. For plasmid DNA transfection, the Lipofectamine 3000 transfection reagent (Invitrogen) was used as per the enclosed protocol.

### Generation of miR-20a knocked-down stable cell lines

MiR-20a was stably knocked down by transducing Huh7 cells with lentivirus miR-20a tough decoy virus particles (Sigma Aldrich). The lentiviral vector also carries a puromycin resistance gene. Huh7 cells were transduced with miR-20a tough decoy lentivirus using an MOI of 20. 48 h after transduction, positively transduced cells were selected using 2ug/mL puromycin for one week. The cells were cultured and aliquots were stored in liquid Nitrogen following verification of miR-20a-5p knockdown by qPCR.

### Generation of the AAV-miR-20a-TuD adeno-associated vector

We generated the AAV-20a-TuD viral plasmid by excising the U6-miR-20a-TuD fragment present in the TRC2-pLKO-U6-20a-TuD-puro plasmid (Sigma Aldrich) and cloning it into a pDS-AAV-TTR plasmid. The resulting sequence-verified plasmid was functionally validated in vitro by transfecting Huh7 cells, followed by qPCR to confirm miR-20a knockdown. Adequate plasmid amounts were then obtained by plasmid megapreps (Endo-free megaprep kit, Qiagen) for subsequent virus production.

### Adeno-associated virus serotype 8 (AAV8) production

HEK293T cells were grown in complete DMEM (4.5 g/ml glucose) medium supplemented with 10% heat inactivated FBS, 1% penicillin/Streptomycin and 1% L-Glutamine. 3*10^6^ cells were seeded per flask in T225cm^2^ flasks in an antibiotics-free medium. 48-hours post-seeding, the culture medium was replaced with antibiotics-free transfection medium and each flask was co-transfected with 25 µg of pDP8.ape plasmid (Plasmid Factory, Bielefeld, Germany) with either of AAV-Control plasmid or AAV-20a-TuD plasmid using CaCl2 transfection method. Media was changed 6-hours later. Cells were harvested 72-hours after transfection, and benzonase treatment was performed, followed by Cesium chloride (CsCl) gradient ultracentrifugation. The virus was collected following ultrafiltration, aliquoted and stored in -80°C. QPCR using primers targeting the TTR promoter was performed to determine viral titers.

### Western blot

Protein expression was determined by western blots. Protein was extracted from cells or tissues using a cell lysis cocktail made with 1x Cell lysis buffer (Cell Signaling Technology), protease inhibitor (Roche) and HALT phosphatase inhibitor (ThermoFisher Scientific). Protein concentration was determined using the Pierce^TM^ BCA Protein Assay Kit (ThermoFisher Scientific). 40 μg of protein from each sample was loaded onto TruPAGE Precast Gels 4-12% (Sigma) and then a wet transfer onto nitrocellulose membrane (Immobilon) was performed. This was followed by blocking with 1x tris-buffered saline with Tween 20 (TBS-T) containing 5% non-fat dry milk powder (Carl Roth), for 1 h at room temperature (RT). Respective primary antibodies were diluted as per the manufacturer’s recommendations and incubated with the membrane, overnight at 4 °C. The membrane was washed multiple times with TBS-T, and incubated at RT for 1 h with a suitable secondary antibody, usually at a dilution of 1:10000 in TBS-T with 5% non-dry fat milk. The membranes were then incubated with Pierce^TM^ ECL Western Blotting Substrate (ThermoFisher) and blots were developed either in a dark room, on a Hyperfilm (Kodak) using developer and fixer (Adefo), or in the Invitrogen iBright Imaging System (Thermo Fisher Scientific). The primary antibodies used for western blots are vinculin (Sigma Aldrich, #V9131), Cytochrome c (CST, #11940; ABCAM, #ab133504), APAF1 (CST, #8969), Caspase 9 (CST, #9508), and BCL-xl (CST, #2764 P). The secondary antibodies, goat anti-rabbit (ABCAM, ab6112) and goat anti-mouse (Santa Cruz, SC-2055), were used.

### In vitro gain- and loss-of-function assays

#### Cell proliferation assays

Cell proliferation and viability were analysed with the WST-1 assay (Roche) as per the manufacturer’s protocol. 1.5 × 10^5^ cells were seeded per well in 6-well plates and 3 × 10^4^ SNU-182 cells were seeded in 24-well plates. The following day, media was replaced with 400 μL of WST-1 reagent diluted 1:10 in media. Cells were then incubated for 2 h in the 37 °C cell culture incubator. Supernatants were transferred to 96-well plates in multiple replicates and absorbance was measured at 450 nm and at 640 nm (reference) using the Synergy™ 2 Multi-Mode Microplate Reader (BioTek, Agilent). Additionally, Click-iT Plus EdU Alexa Fluor 488 kit (ThermoFisher) was used to study cell proliferation as per the provided manufacturer’s protocol, followed by flow cytometry. EdU-positive cells are identified as proliferating cells. FlowJo software was used to analyse the data.

#### Wound healing assay

Cell migration was analysed using two-chambered cell culture inserts (Ibidi). 1.5 × 10^4^ cells were seeded per chamber and the rate of cell migration was monitored over 72 h to determine how fast the cells migrate to close the 500μm space between the two chamber sections following removal of the insert. ImageJ was used to analyse pictures taken with a bright field microscope at the experimental endpoints.

#### Colony forming assay

Soft agar colony forming assays were performed as per an established standard protocol in our laboratory. Briefly, 2% low melt agar (NuSieve GTG Agarose) in DMEM culture media without FBS or P/S (ThermoFisher) was prepared and diluted to 1% with complete DMEM media. The bottom layer consisting of 2 ml of this diluted 1% low melt agar per well in 6-well plates, was then allowed to solidify. 1 × 10^4^ Huh7 cells or 2 × 10^4^ SNU-182 cells suspended in 0.5% (final dilution) low melt agar in complete media were then seeded per well over the bottom layer and allowed to set. Then, each well was covered with 1 ml of complete media to prevent drying. The cells were cultured for two to four weeks after which pictures were taken under the microscope, and colonies were counted using ImageJ software.

#### LT2M HCC mouse organoids assay

Briefly, LT2M cells were transfected with 100 nM of either a miR-20a-5p inhibitor or a scramble control inhibitor (MIRIDIAN, Dharmacon) using Dharmafect 4 as the transfection reagent. 48 h post transfection, the cells were gently harvested, washed to remove media, counted, and diluted to a 150 × 10^4^cells/ml and mixed with Cultrex™ Reduced Growth Factor Basement Membrane Extract, Type 2, PathClear™ (BME), at a 1:2 ratio. Finally, 2.5 × 10^4^ cells were used per well of a 24-well plate. After the BME solidified, 500ul organoid culture medium (Supplementary Table S2) was added to each well and the plate was returned to the cell culture incubator. Developed organoids were clearly visible on day 6 after seeding. These were then carefully isolated using a Cell Recovery Solution (Corning) and very carefully washed with cold 1x PBS and transferred to wells of a 12-well plate in cold 1x PBS for further examination and analyses.

#### Apoptosis detection

The Annexin V Apoptosis detection kit with 7AAD (Biolegend) was used. 1.5 × 10^5^ cells were seeded per well in a 6-well plate. 48-hours post-seeding, the cells were harvested and stained with Annexin V and 7AAD. Cells were then analysed by flow cytometry using LSRII (BD Biosciences), and results were analysed using the FlowJo software.

#### Caspase 3/7 activity assay

1 × 10^4^ cells were seeded per well in a 96-well plates and the Caspase-Glo 3/7 Assay activity kit (Promega) was used to determine caspase 3/7 activity as per the manufacturer’s enclosed protocol.

### Luciferase assay

The sense and antisense fragments of the human and mouse CYCS 3’UTR regions containing either the wild-type or the mutated (human)/deleted (mouse) miR-20a binding sites were synthesized (Sigma). The respective sense and its corresponding antisense strands were hybridised and cloned into the pmirglo Dual-Luciferase vector (Promega) at the SacI and XbaI sites. 1 × 10^4^ cells were seeded per well in a 96-well plate and the cells were co-transfected with the pmirglo vector or the pmirglo-Cyt c vector and the miR-20a mimic using the Dharmafect Duo (Dharmacon, Horizon Discovery) transfection reagent. The Dual-Glo luciferase assay kit (Promega) was used to measure luciferase activity, 48 h post-transfection. Luminescence was the readout, measured using the GloMax® Explorer plate reader.

### Metabolic assays

Metabolic assays were performed by seeding 1 × 10^4^ Huh7_LV-Control or LV-20a-TuD cells per well in the appropriate 96-well plates for either luminescence or fluorescence read-outs.

#### Glucose uptake assay

Using the Glucose Uptake-Glo^TM^ assay kit (J1341, Promega), glucose uptake was determined following the provided manufacturer’s instruction. Luminescence signal was measured using a Synergy 2 Multi-Mode plate reader and the Gen5 software.

#### Lactate assay

The Lactate-Glo^TM^ kit (J5021, Promega) was used to separately measure extracellular and intracellular lactate. 1 × 10^4^ LV-Control or LV-20a-TuD cells were seeded per well in a 96-well plates and the enclosed manufacturer’s protocol was followed. Luminescence was measured using a Synergy 2 Multi-Mode plate reader and the Gen5 software.

#### Extracellular acidification rate assay (ECAR)

ECAR was performed to determine the rate of glycolysis in cells using the Glycolysis Assay kit (Abcam). For this assay, 4 × 104 LV-Control or LV-20a-TuD cells were seeded per well in a 96-well plate. The assay was then performed based on the manufacturer’s protocol provided in the kit, and fluorescence intensity was measured using the BioTek SynergyTM H1 Multi-Mode Microplate Reader.

#### Extracellular oxygen consumption assay (OCR)

1 × 10^4^ Huh7_LV-Control or LV-20a-TuD were seeded per well in a black 96-well plate. The OCR assay (Abcam) was performed following the provided manufacturer’s instruction, and fluorescence readout was measured using a microplate reader.

#### ATP Detection Assay

ATP levels in cells were detected using the Luminescent ATP Detection Assay Kit (Abcam 113849). 1 × 10^4^ LV-Control and LV-20a-TuD cells were seeded per well in a 96-well plate in complete medium (DMEM with 4.5 g/L glucose, +P/S, +10% FBS). The following day, a subset of wells with LV-20a-TuD cells were transfected with 50 nM of miR-20a mimic (Dharmacon, Horizon Discovery) while the LV-Control cells and the remaining subset of wells with LV-20a-TuD cells were transfected with 50 nM of miR-Scramble. Media was changed 12 h post transfection and ATP detection assay was performed 48 h post-transfection as per the enclosed protocol. Luminescence was measured using the SynergyTM 2 Multi-Mode Microplate Reader. In a separate experiment, LV-Control and LV-20a-TuD cells were seeded as described above. The following day, a subset of wells with LV-20a-TuD cells were transfected with 50 nM of either negative ON-TARGETplus non-targeting pool control (Scr siRNA) or the CYCS siRNA (Dharmacon, Horizon Discovery) while the LV-Control cells and the remaining subset of wells with LV-20a-TuD cells were untransfected. As described above, ATP detection assay was performed 48 h post transfection using the GloMax® Explorer plate reader.

### Histology and staining

#### Hematoxylin and eosin (H&E), TUNEL and Ki67 staining

Formalin-fixed paraffin embedded 5μm tissue sections were used for all histological staining. To examine changes in tumor morphology and histology, a well-established standard H&E staining protocol was used. To stain for apoptotic cells, TUNEL staining was performed as per the Apoptag Peroxidase In Situ Apoptosis Detection Kit (Millipore). Ki67 immunohistochemical staining was performed to detect proliferating cells using well-established protocols in our laboratory with the Ki67 primary antibody (Ki67, SP6, Catalog # MA5-14520, ThermoFisher).

#### Immunofluorescence (IF) staining

Immunofluorescence staining for Cytochrome c was performed using a well-established protocol in our laboratory on Huh7 LV-Control and LV-20a-TuD cells, as well as on LV-20a-TuD cells treated with either the Scramble control or the miR-20a-5p mimic. Briefly, 48 h following mimic treatment, cells were washed in 1X PBS and fixed in 4% paraformaldehyde for 15 min. The cells were then permeabilized in a permeabilization solution (0.3% Triton-X in 1X PBS). Following 1x PBS washes, cells were blocked for one hour in a 1X PBST blocking buffer containing 10% Goat serum. The cells were then incubated overnight at 4 °C with the Cytochrome c primary antibody (Abcam, ab133504) diluted 1:100 in an antibody dilution buffer containing 0.3% Triton X in 1X PBST and 1% BSA. The following day, cells were incubated for one hour in secondary antibody (ThermoFisher Scientific, A-11008) diluted 1:750 in 1% BSA in 1X PBST, and then counterstained with DAPI, and visualized under a fluorescence microscope.

#### Statistical analyses

Test of significance was performed using two-tailed Student’s *t* test for comparison between two groups. Error bars represent ± standard error mean (SEM). Sample size and *p* values are included in figure legends. *P* < 0.05 was considered significant. Survival studies were analysed by Kaplan-Meier analyses.

### Supplementary information

Supplemental_Information_Figures_Legends_and_Tables

Original Western blots

## Data Availability

All data relevant to our study are included in the main article or uploaded as supplementary data. Additionally, miRNA and mRNA expression array data have been submitted to GEO.
