# Supplementary material for: MicroRNA miR-20a-5p targets CYCS to inhibit apoptosis in hepatocellular carcinoma
Source: Cell Death Dis. 2024 Jun 27;15(6):456. doi: 10.1038/s41419-024-06841-0 (PMC11211328; doi:10.1038/s41419-024-06841-0)
Supplement: Supplementary file 1 — Supplemental_Information_Figures_Legends_and_Tables [file 41419_2024_6841_MOESM1_ESM.pdf]

SUPPLEMENTARY FIGURES, LEGENDS AND TABLES

Supplementary Figure S1

A.

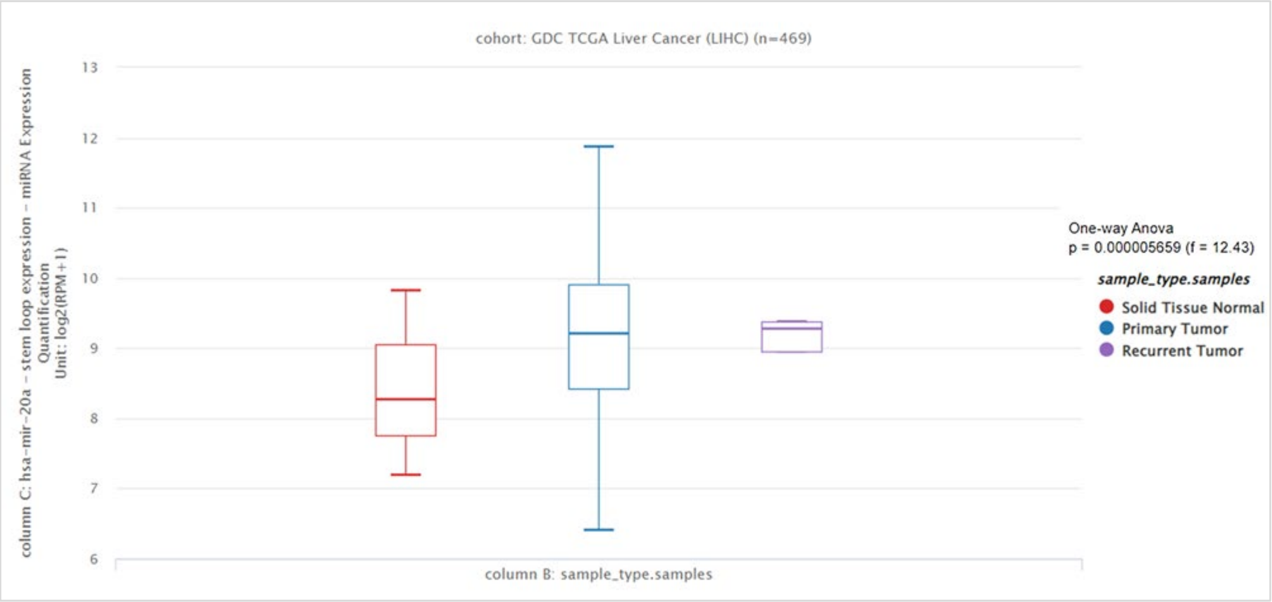

B.

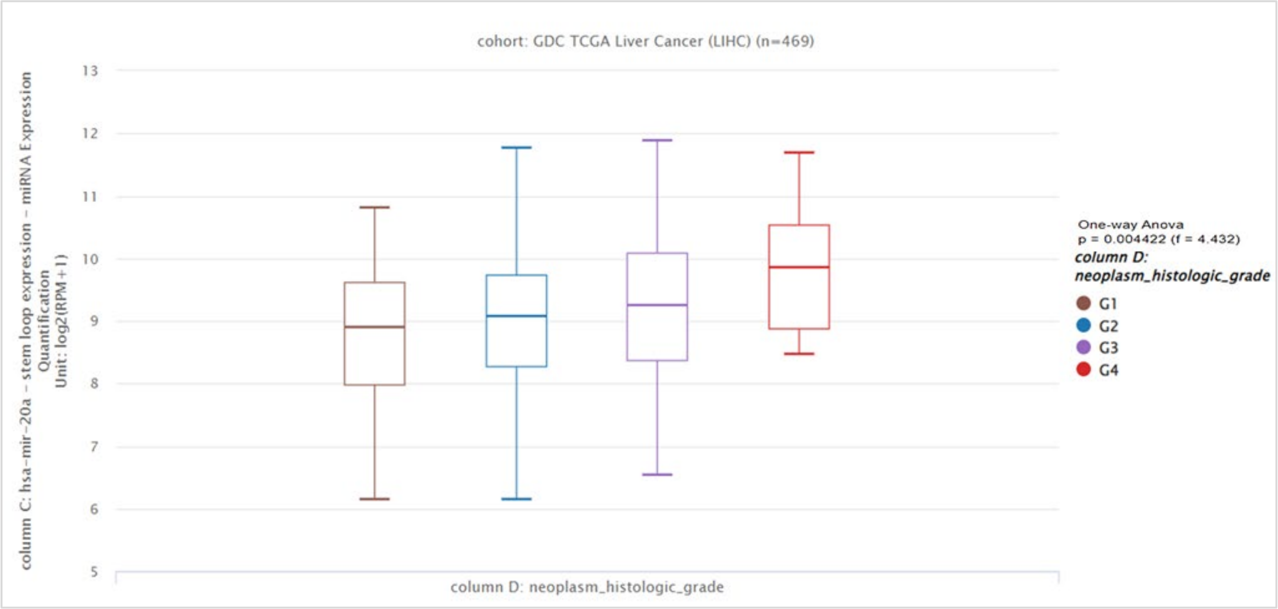

C.

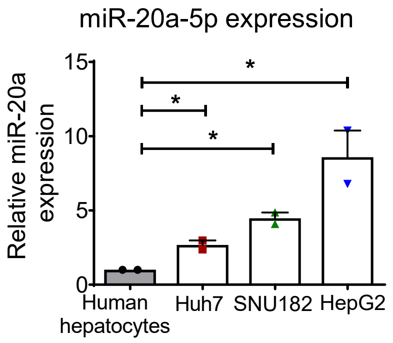

D.

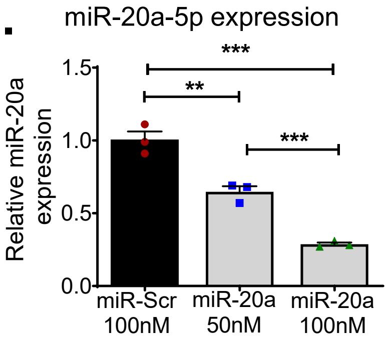

Supplementary Figure S1. MiR-20a-5p in human HCCs. Graphs from the TCGA-LIHC dataset showing **A.** higher miR-20a-5p expression in primary tumors compared to solid tissue normal samples (One-way ANOVA, p=0.000005659) and **B.** increasing miR-20a-5p expression with higher histological tumor grades G1<G2<G3<G4 (One-way ANOVA, p=0.004422). **C.** qPCR quantification of miR-20a expression in the human HCC cell lines, Huh7, SNU182 and HepG2 relative to normal human hepatocytes. *, $P < 0.05$. **D.** Determination of the optimum concentration of miR-Inhibitor for targeted knock-down of miR-20a in Huh7 cells (n=3). **, $P < 0.008$; ***, $P < 0.0004$. The value of n equals the number of wells seeded with cells per treatment group. Unless specified, data are presented as mean ± SEM. *P* values were determined by two-tailed Student's *t* test.

Supplementary Figure S2

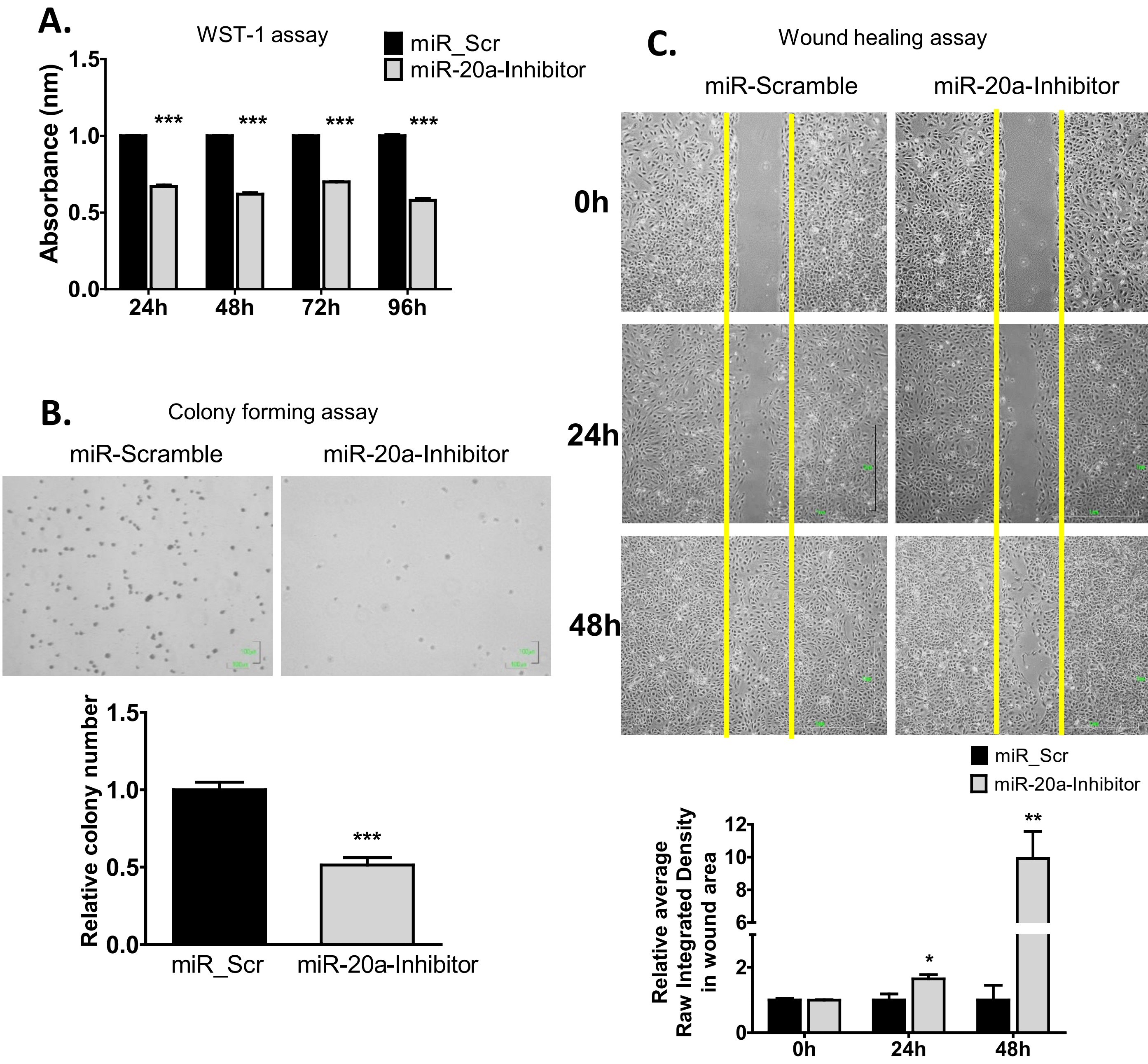

Supplementary Figure S2. MiR-20a-5p inhibition attenuates cancer hallmarks in SNU182 cells. Analysing the effects of miR-20a-5p inhibition in SNU182 hepatoma cells transfected with either Scr or miR-20a-5p inhibitor. **A.** WST1 (n=10 independent biological replicates/treatment group/time point) **B.** Soft agar colony formation (n=6 independent biological replicates/treatment group) and **C.** Wound healing (n=5 independent biological replicates/treatment group) assays showed a clear inhibition of cancer hallmarks following miR-20a-5p knockdown. *, $P < 0.05$; **, $P < 0.005$; ***, $P < 0.0001$. Data are presented as mean ± SEM. P values were determined by two-tailed Student's t test.

Supplementary Figure S3

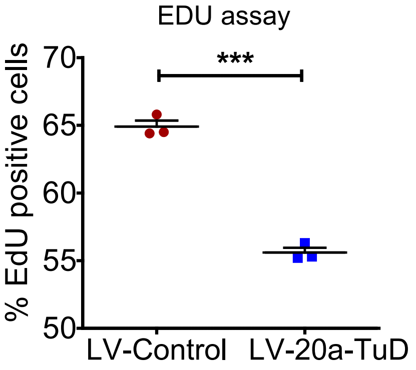

Supplementary Figure S3. Effect of miR-20a-5p knockdown on cell proliferation. Click-iT EdU assay shows decreased cell proliferation in LV-20a-TuD cells compared with LV-Control cells (n=3 independent biological replicates per group). ***, $P < 0.0001$.

Supplementary Figure S4

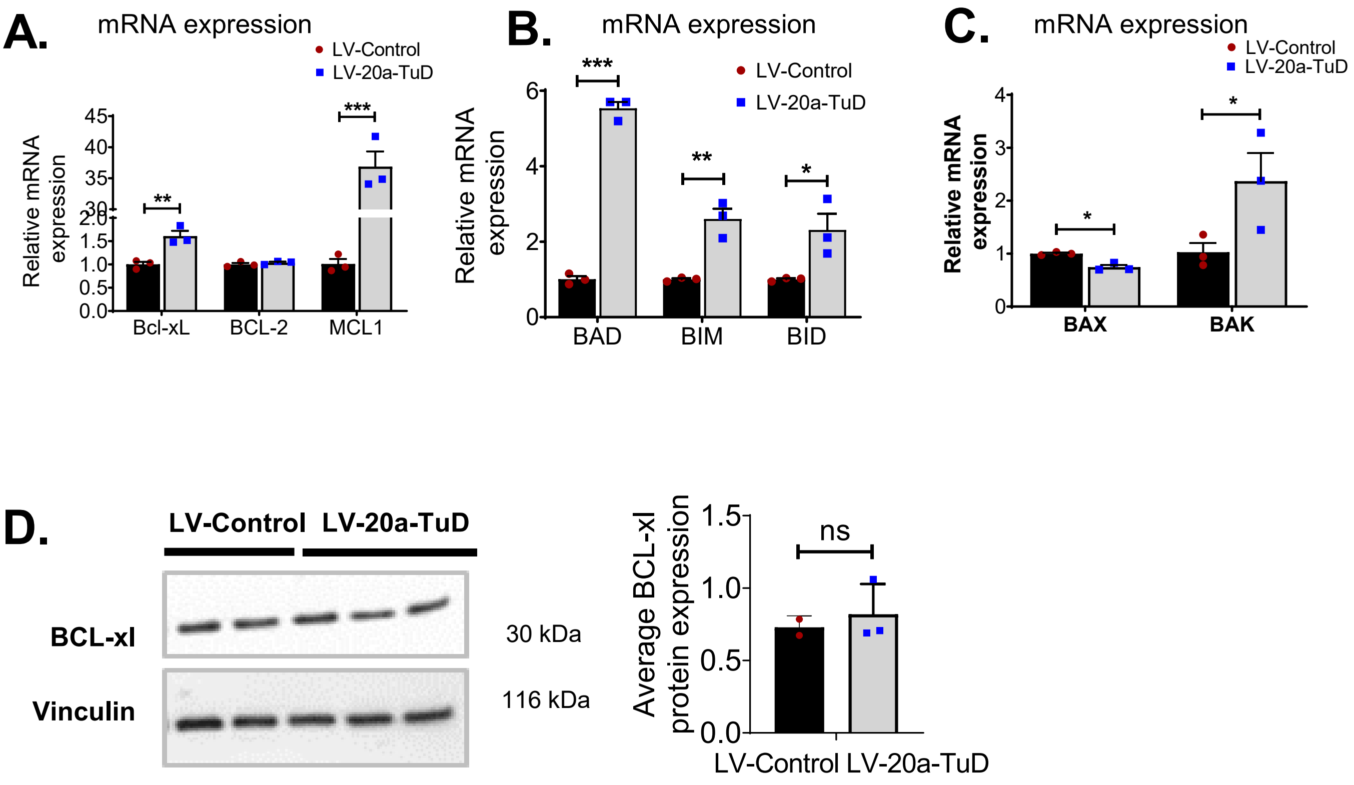

Supplementary Figure S4. A. qPCR quantification to determine expression of the BCL-2 family anti-apoptotic genes BCL-xL, BCL-2 and MCL1 (n=3 independent biological replicates /gene/group). **, *P* < 0.01; ***, *P* < 0.0001. **B.** Determination of the mRNA expression of key BCL-2 pro-apoptotic genes BAD, BIM and BID via qPCR. (n=3 independent biological replicates /gene/group).*, *P* <0.01; **, *P* < 0.001; ***, *P* < 0.0001. **C.** qPCR to determine mRNA expression of apoptosis effectors BAX and BAK (n=3 independent biological replicates /gene/group). *, *P* < 0.05. LV-20a-TuD cells are compared to LV-Control cells. **D.** Western blot shows no significant increase in BCL-xl protein expression in LV-20a-TuD cells compared to LV-Control cells (LV-Control: n=2; LV-20a-TuD n=3 independent biological replicates). Data are presented as mean ± SEM. *P* values were determined by two-tailed Student's *t* test.

Supplementary Figure S5

A.

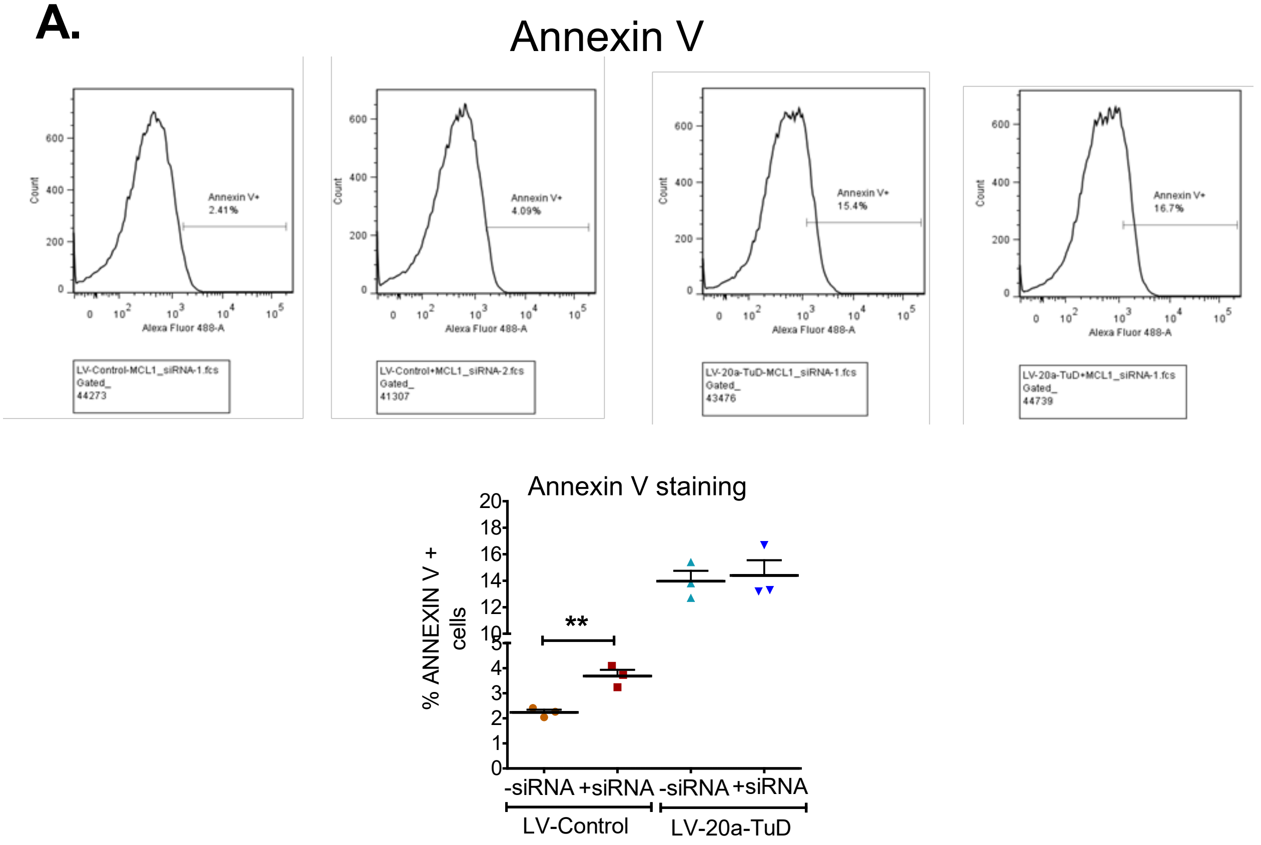

B.

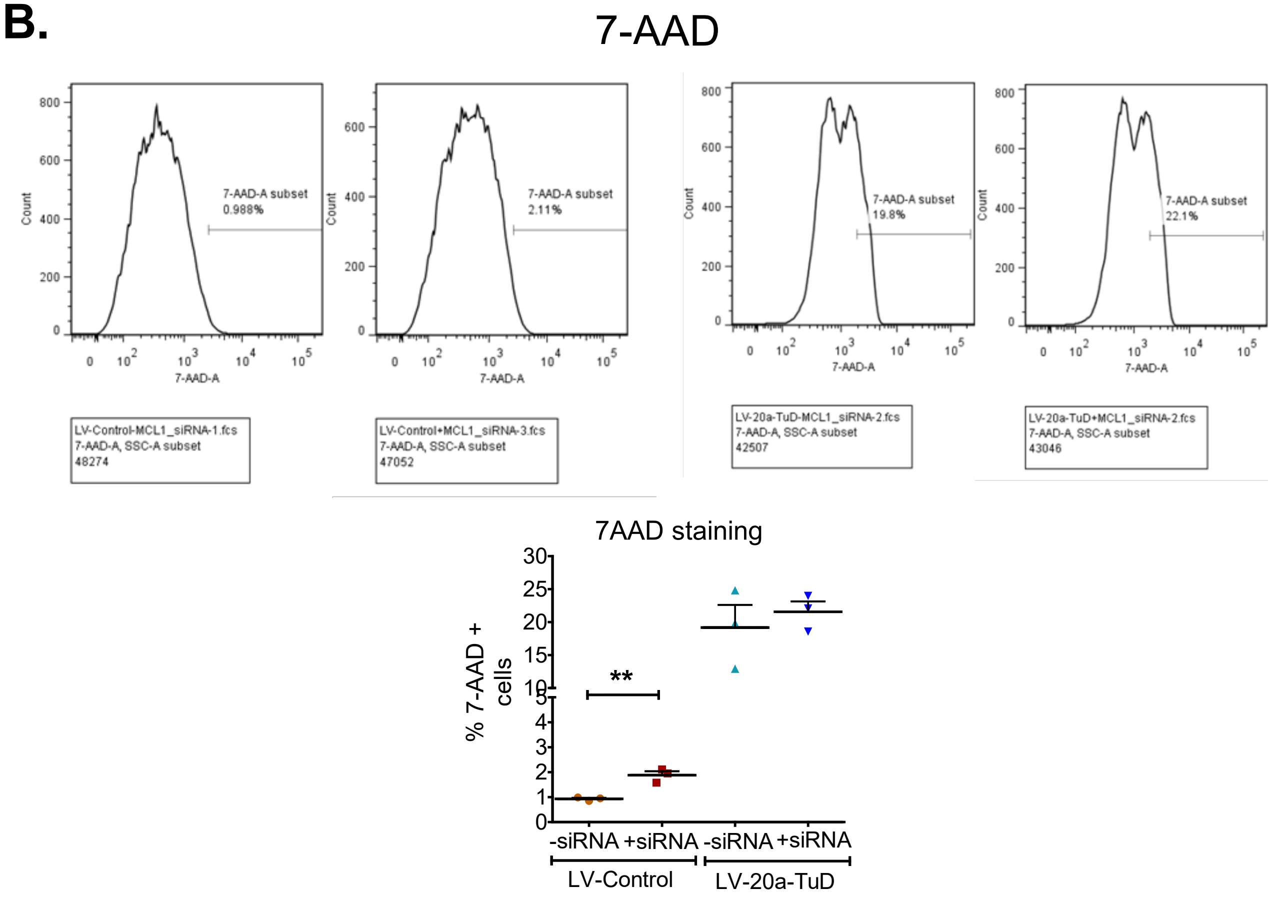

Supplementary Figure S5. Representative histograms and graphs from flow cytometry analyses showing **A.** ANNEXIN V positive and **B.** 7-AAD positive LV-Control and LV-20a-TuD cells transfected with MCL1 siRNA and siRNA-Negative control (n=3). The value of n equals the number of independent biological replicates per treatment group. Data are presented as mean ± SEM. *P* values were determined by two-tailed Student's *t* test. ANNEXIN V: **, *P* = 0.005. 7-AAD: **, *P* = 0.004.

Supplementary Figure S6

A.

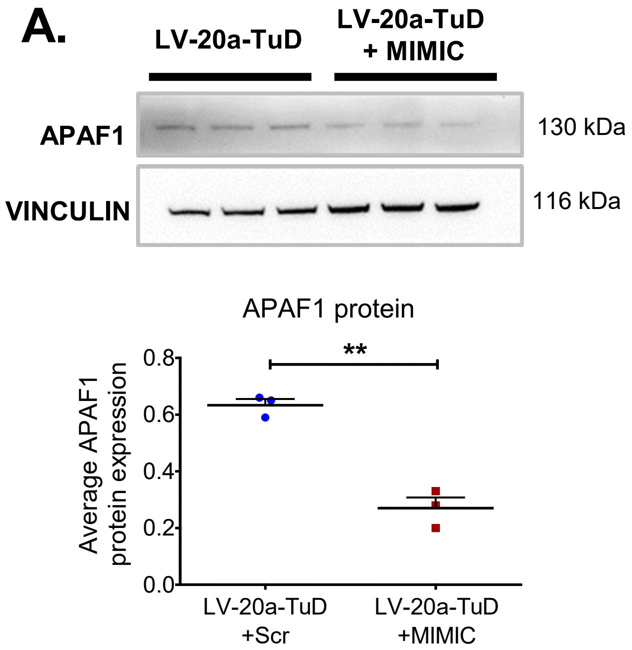

B.

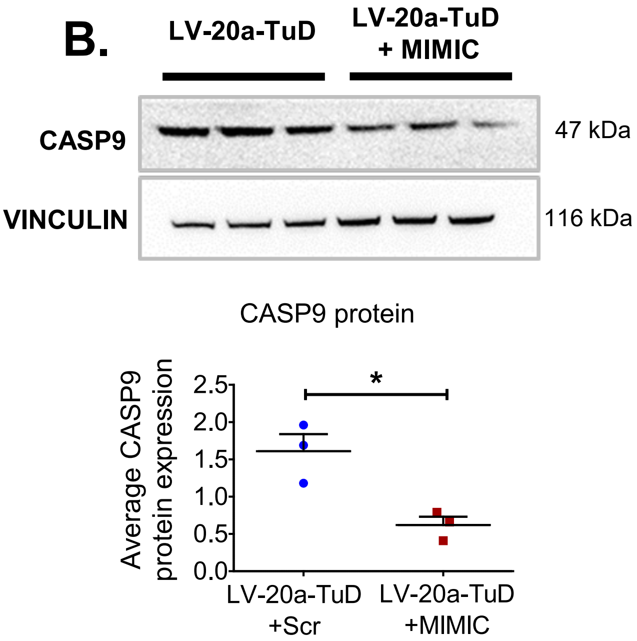

Supplementary Figure S6. Western blots show significant downregulation of protein expression of **A.** APAF1 and **B.** Caspase 9 (CASP9) in LV-20a-TuD-MIMIC treated cells compared to scramble treated LV-20a-TuD cells (n=3). The value of n equals the number of independent biological replicates per treatment group. Data are presented as mean ± SEM. *P* values were determined by two-tailed Student's *t* test. APAF1: **, $P < 0.002$. CASP9: *, $P < 0.02$.

Supplementary Table S1

A. List of qPCR SYBR Green Primers

SYBR Green Primers	Forward Primer	Reverse Primer
hsa/mmu ß-actin	GATCAAGATCATTGCTCCTCCT	TGATCCACATCTGCTGGAAG
hsa-BIM	CAGTGCAATGGCTTCCATG	AATACCCTCCTTGCATAGTAAGC
hsa-BAX	CGAGTGGCAGCTGACATG	GTGCACAGGGCCTTGAG
hsa-BAD	GGACTCCTTTAAGAAGGGACTTC	CCACCAGGACTGGAAGACTC
hsa-BID	CGGATTCTGTCGCCACTG	ACCGTTGTTGACCTCACAGTC
hsa-BCL-XL	GTAGACAAGGAGATGCAGGTATTG	TCCACAAAAGTATCCCAGCC
hsa-MCL1	GCCTTCCAAGGCATGCT	CAGTTTGTTACGCCGTCG
hsa-BCL2	GTCAACCGGGAGATGTCG	TCCACAAAGGCATCCCAG
hsa-BAK	GCTTCTGAGGAGCAGGTAGC	CCATGGTGCTGCTAGGTTG
hsa-CYCS	TCTCCATGGTCTCTTTGGGC	ATCCTCTCCCCAGATGATGC
mmu-CYCS	AGGCTGCTGGATTCTCTTACAC	GTTCCAGGGATGTACTTTTTGG
hsa-Casp3	TGCATACTCCACAGCACCTG	TCAAGCTTGTCGGCATACTG
hsa-Casp7	TGTGCTTCTTATGTTACCCAGAT	CTTGCACAAACCAGGAGCCTC
AAV-TTR-Titration	TCAGCTTGGCAGGGATCAG	GACGGCTTCTCCTGGTGAAG
U6-20a-TuD-For	GCATATACGATACAAGGCTG	

B. qPCR TaqMan probes

TaqMan Probe	Company	Catalogue Number	Assay ID
U6 snRNA TaqMan control	ThermoFisher Scientific	4440887	001093
hsa/mmu-miR-20a	ThermoFisher Scientific	4427975	000580

Supplementary Table S2

Organoid culture medium

**Stock concentration**	**Final concentration**	**Volume**
AdDMEM/F12		50mL
HEPES 1M	10mM	0.5mL
GlutaMax 100X	1X	0.5mL
Penicillin-Streptomycin100X	1X	0.5mL
RSPO 50µg/mL	150ng/mL	150µl
B27 50X	1X	1mL
N-acetylcysteine (Sigma) 500mM/mL	1.25mM/mL	125µl
Nicotinamide (Sigma)1M/mL	10mM/mL	0.5mL
gastrin (Sigma) 500µg/mL	20ng/mL	2µl
CHIR99021 10nM/mL	3µM/mL	15µl
EGF (Peprotech) 100µg/mL	5ng/mL	5µl
HGF (Peprotech) 100µg/mL	5ng/mL	5µl
FGF7 (Peprotech) 100µg/mL	5ng/mL	5µl
FGF10 (Peprotech) 100µg/mL	5ng/mL	5µl
A83-01 5mM/mL	1µM/mL	10µl
Y27632 3mg/mL(10mM/mL)	3µg/mL(10µM/mL)	50µl
