# Supplementary material for: MicroRNA miR-20a-5p targets CYCS to inhibit apoptosis in hepatocellular carcinoma
Source: Cell Death Dis. 2024 Jun 27;15(6):456. doi: 10.1038/s41419-024-06841-0 (PMC11211328; doi:10.1038/s41419-024-06841-0)

WESTERN BLOTS

NEW_REVISED_Figure 3B : CYCS and Vinculin. LV_Control and LV_20a-TuD cells.

Figure 3B

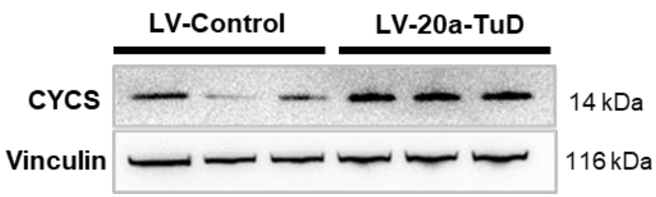

Original blots

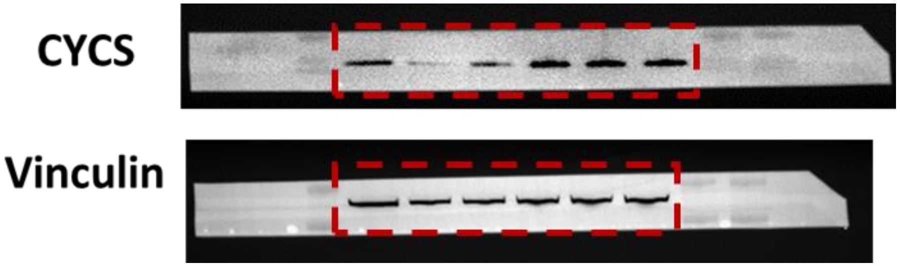

REVISED_Figure 4C : CYCS and Vinculin. LV_20a-TuD cells + Scr or miR-20a-mimic.

Figure 4C

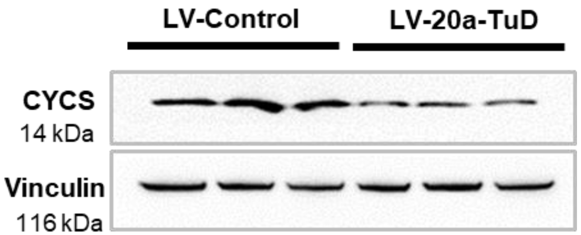

Original blots

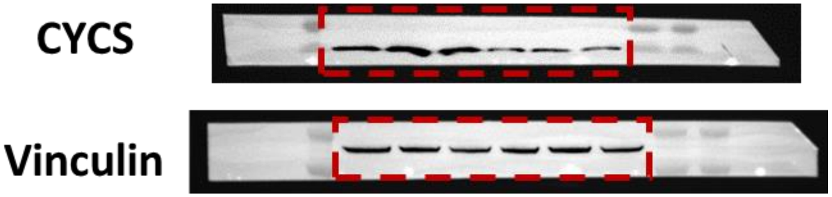

Figure 7E

CYCS and Vinculin in tumor-bearing liver tissues from AAV-20a-TuD and AAV-Control treated LT2/RAS mice.

Figure 7E

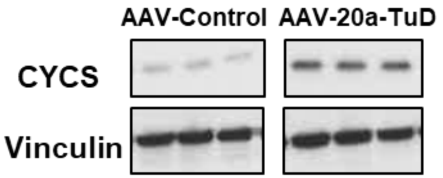

Original blot/film

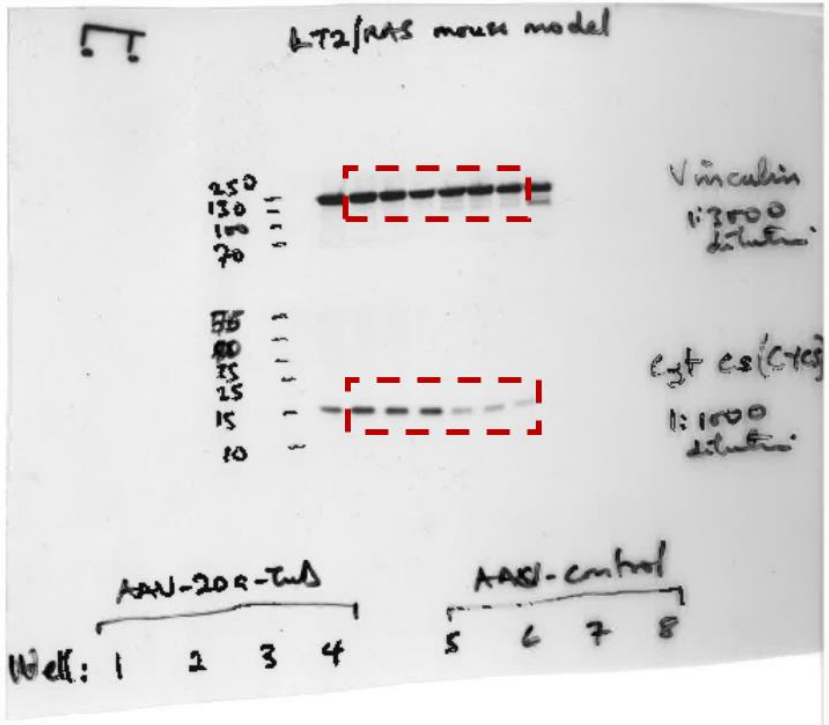

REVISED_Supplementary Figure S4D

BCL-xl and Vinculin. LV_Control and LV_20a-TuD cells.

Supplementary Figure S4D

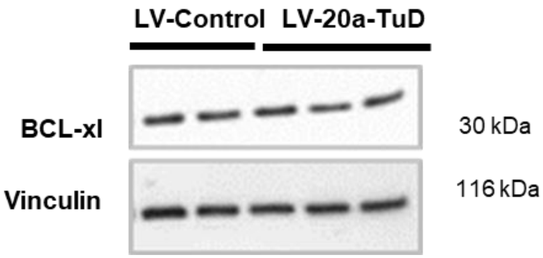

Original blots/films

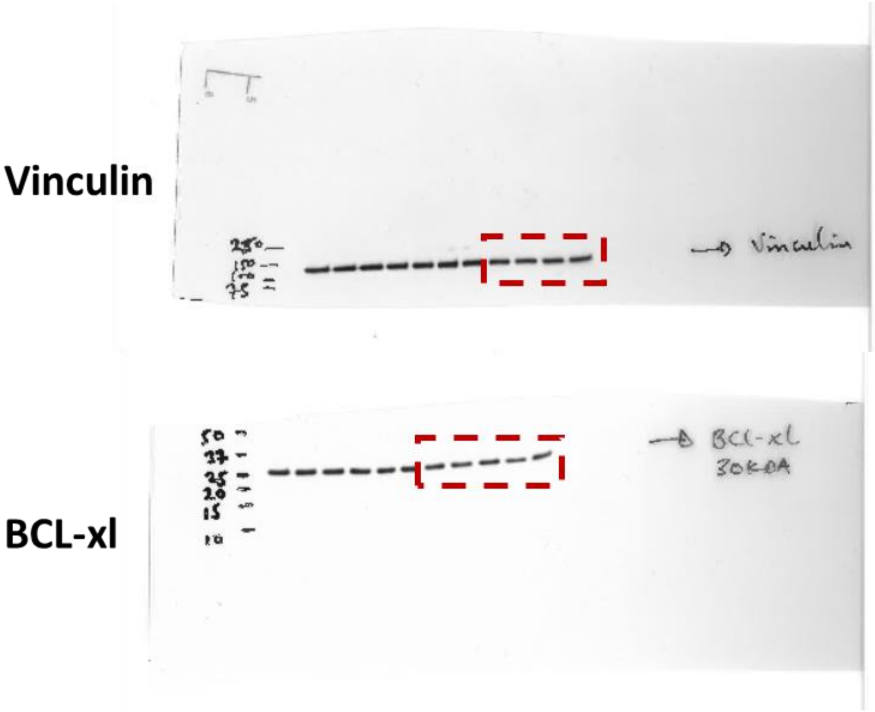

REVISED_Figure S6A and S6B :

S6A : APAF1 and Vinculin. S6B: Casp9 and Vinculin.

LV_20a-TuD cells + Scr or miR-20a-mimic.

S6A.

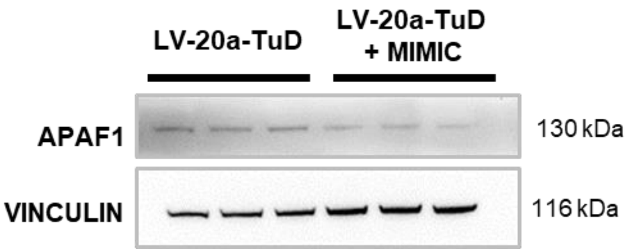

Original blots

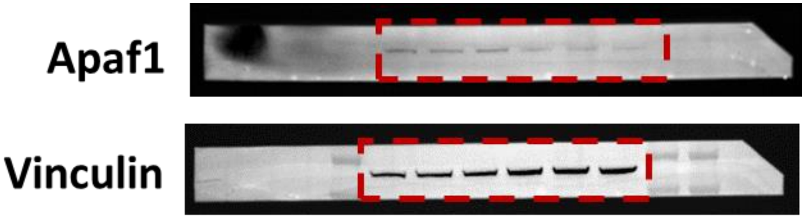

S6B.

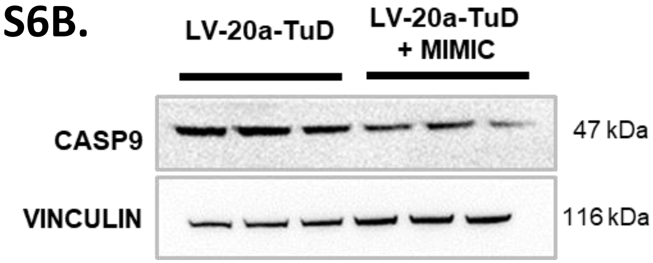

Original blots

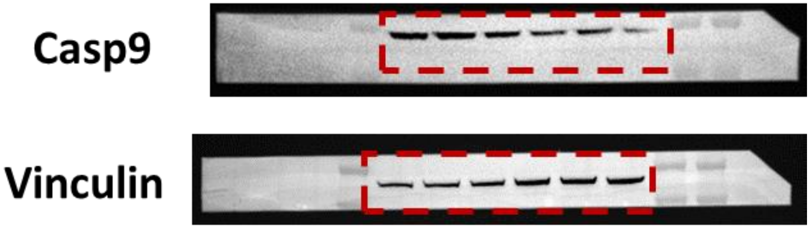

Supplement: Supplementary file 2 — Original Western blots [file 41419_2024_6841_MOESM2_ESM.pdf]
